# Influence of Outliers on Accuracy Estimation in Genomic Prediction in Plant Breeding

**DOI:** 10.1534/g3.114.011957

**Published:** 2014-10-01

**Authors:** Sidi Boubacar Ould Estaghvirou, Joseph O. Ogutu, Hans-Peter Piepho

**Affiliations:** Biostatistics Unit, Institute of Crop Science, University of Hohenheim, 70599 Stuttgart, Germany

**Keywords:** accuracy estimation, genomic prediction, outliers, predictive accuracy, heritability, GenPred, shared data resource

## Abstract

Outliers often pose problems in analyses of data in plant breeding, but their influence on the performance of methods for estimating predictive accuracy in genomic prediction studies has not yet been evaluated. Here, we evaluate the influence of outliers on the performance of methods for accuracy estimation in genomic prediction studies using simulation. We simulated 1000 datasets for each of 10 scenarios to evaluate the influence of outliers on the performance of seven methods for estimating accuracy. These scenarios are defined by the number of genotypes, marker effect variance, and magnitude of outliers. To mimic outliers, we added to one observation in each simulated dataset, in turn, 5-, 8-, and 10-times the error SD used to simulate small and large phenotypic datasets. The effect of outliers on accuracy estimation was evaluated by comparing deviations in the estimated and true accuracies for datasets with and without outliers. Outliers adversely influenced accuracy estimation, more so at small values of genetic variance or number of genotypes. A method for estimating heritability and predictive accuracy in plant breeding and another used to estimate accuracy in animal breeding were the most accurate and resistant to outliers across all scenarios and are therefore preferable for accuracy estimation in genomic prediction studies. The performances of the other five methods that use cross-validation were less consistent and varied widely across scenarios. The computing time for the methods increased as the size of outliers and sample size increased and the genetic variance decreased.

Genomic selection, the prediction of genomic breeding values using molecular markers spanning the entire genome (Meuwissen *et al.* 2001; Piepho 2009; Whittaker *et al.* 2000), is becoming a widespread practice in plant and animal breeding because of recent advances in high-throughput marker technologies and decreasing costs of genotyping (Meuwissen *et al.* 2001; Bernardo and Yu 2007; Goddard and Hayes 2007).

Accordingly, it is important to assess the performance of procedures for estimating predictive accuracy in genomic prediction studies under a variety of realistic scenarios likely to be encountered in plant breeding research and practice. In particular, the performance of methods for estimating predictive accuracy in genomic prediction studies may be expected *a priori* to be adversely affected by outliers. As a result, outliers are potentially a highly relevant problem in accuracy estimation in genomic prediction (Via *et al.* 2012; Heslot *et al.* 2013). It is therefore crucial to evaluate and rank contending methods for accuracy estimation in genomic prediction in terms of their estimated predictive accuracies and degree of robustness to outliers in phenotypic datasets. Accurately conducting such evaluations presupposes knowledge of the underlying true predictive accuracy for use in benchmarking the relative performance of the competing methods with and without outliers. However, for real datasets, the true underlying predictive accuracy is rarely, if ever, known.

Outliers can potentially have devastating effects on estimated quantities of interest (Atkinson 1985; Belsley *et al.* 2005), but their effects on accuracy estimation in genomic prediction studies in plant breeding thus far have not been evaluated. In plant breeding, outliers can arise from an outlying observation, environment, or year in a series of trials. Although many statistical procedures have long been developed for identifying outliers, successful identification and removal of true outliers from data remain a very challenging task to date, especially in high-dimensional data typically used in genomic prediction, and may even have undesirable consequences (Cook 1977; Filzmoser 2005; Cerioli 2010; Cerioli and Farcomeni 2011). Key complications in outlier detection and removal include masking or swamping of true outliers rendering them inconspicuous, difficulties associated with differentiating mild outliers from regular observations, and reduction in sample size and potentially adverse impact on the assumed distribution theory and, hence, estimated variance of removing outliers (Lourenço and Pires 2014). Because data cleaning procedures are imperfect and do not always succeed in detecting and eliminating all true outliers, it is important to assess the performance and robustness of methods for accuracy estimation in the presence of outliers in genomic prediction studies.

Here, we examine the influence of outliers on the performance and robustness of seven methods for estimating the accuracy of heritability and prediction accuracy in genomic prediction studies in plant breeding. To mimic outliers, we added to only one randomly chosen observation in each of the 1000 phenotypic datasets simulated for each scenario, in turn, each of the three multiples of the SDs of the two residual error variances used to simulate the small and the large datasets. The influence of the outliers on the performance of each of the seven methods was then evaluated by using each method to estimate predictive accuracy based on matched datasets with and without outliers. The deviation of the predictive accuracy estimated using the dataset without outliers from the simulated true predictive accuracy was used as the gold standard against which to evaluate the corresponding deviation in the predictive accuracy estimated using the same dataset with outliers.

## Materials and Methods

This article expands on the article by Estaghvirou *et al.* (2013) by contaminating the phenotypic datasets simulated for that article with outliers to assess their influence on the methods for accuracy estimation in genomic prediction. Otherwise, both articles use the exact same seven genomic prediction methods, simulation models, real maize, and SNP marker datasets to parameterize the simulation models and, hence, the same simulated phenotypic datasets. The new aspects of the methods concerning the outliers are thus highlighted here, whereas aspects of the methods that are similar between the two articles and that have already been described more fully in the earlier articles are only briefly summarized. This is particularly the case for the derivations of the equations used to compute heritability by method 4 and to estimate prediction accuracy by methods 5 and 7. Key details of the SAS code (SAS Institute 2014) for implementing all seven methods are described comprehensively in the earlier article and therefore are not reproduced here. The statistics, parameters, and symbols used to denote them in the text are summarized in Supporting Information, Table S1.

More precisely, we simulated 1000 datasets for each of 10 scenarios to evaluate the magnitude and direction of the influence of outliers on estimates of predictive accuracy for seven contending methods for accuracy estimation in genomic prediction. The 10 scenarios were defined by combinations of the number of genotypes (small = 177, large =698), marker effect variances [(small = (0.2019, 0.2019/10), large = (0.005892, 0.005892/10)], and multiples (5, 8, 10 for small datasets; 5, 10 for large datasets) of the SDs of the errors (small = 48.6728, large = 53.8715) used to simulate the phenotypic datasets. Although we showed the performances of methods 5 and 7 to be the best overall of the seven methods in a previous study (Estaghvirou *et al.* 2013), we nevertheless consider all seven methods again here because it is not possible to deduce their likely performances in the presence of outliers based on findings of that study.

### Prediction accuracy

The correlation between the true (g) and predicted ($\hat{g}$) breeding values, called the simulated true accuracy or predictive accuracy, is defined as:

$$r_{g,\hat{g}}=\frac{s_{g,\hat{g}}}{\sqrt{s_{g}^{2}s_{\hat{g}}^{2}}}$$, (1)

where $s_{g,\hat{g}}$ is the covariance between the true and predicted breeding values, $s_{g}^{2}$ is the sample variance of the true genetic breeding values, and $s_{\hat{g}}^{2}$ is the sample variance of the predicted breeding values. Our use of (1) is premised on the assumption that the correlation between the true and predicted breeding values ($r_{g,\hat{g}}$) is the quantity of primary interest to breeders or geneticists.

### Two-stage approach for predicting breeding values

We used a two-stage approach to predict the breeding values (Schulz-Streeck *et al.* 2013). We considered only one trial conducted in one location. In the first stage, we used the model for the observed plot data to estimate the adjusted means *p* for the testcross genotypes

$$p=X_{1}\eta +f,$$ (2)

where *p* is the vector of the adjusted means of the observed phenotypic values, $\eta ={\left(\eta_{1},\ldots ,\eta_{n}\right)}^{T}$ is the vector of the genotypic means from the second stage, $X_{1}$ is the design matrix, and *f* is a matrix containing all the fixed and random (design and error) effects (replicates, blocks, etc.). A more elaborate specification of the two-stage-model, including a more precise description of the terms in model (2), is provided in Piepho *et al.* (2012a). In our case, the residual vector *f* for the simulated data comprises effects for complete replicates, incomplete blocks, and plot error.

In the second stage, the true breeding values (g) were predicted using the adjusted means for the testcross genotypes (but excluding any standard varieties) computed in (2) as the response and the model

$$\hat{\eta }=\varphi +g_{i}+e_{i},$$ (3)

where $\hat{\eta }$ is the adjusted mean of the *i*-th genotype, *φ* is the general mean, $g_{i}$ is the random effect of the *i*-th genotype, and $e_{i}$ is the residual error assumed to be N(0,R). The random vector $g={\left(g_{1},\ldots ,g_{n}\right)}^{T}$ is modeled through a linear regression on the random marker (SNP) effects $u={\left(u_{1},\ldots ,u_{n}\right)}^{T}$ by

g=Zu, (4)

where *Z* is the matrix of the marker covariates and $u∼N\left(0,I\sigma_{u}^{2}\right)$, $I_{p}$ is the *p*-dimensional identity matrix, and $\sigma_{u}^{2}$ is the variance of marker effects. The marker information stored in the matrix $Z_{snp}=\left\{z_{ik}\right\}$ consisted of 275 (AgReliant dataset) or 11646 (KWS Synbreed dataset) SNP markers derived from genotyping all the testcross genotypes. The marker covariate $z_{ik}$ for the *i-th* genotype (i=1,…,n) and the *k-th* marker (k=1,…,q) for biallelic SNP markers with alleles $A_{1}$ and $A_{2}$ was recoded as

$$z_{ik}=\{\begin{array}{cc} 1 & \text{for}\;{\text{A}}_{1}{\text{A}}_{1}\\ -1 & \text{for}\;{\text{A}}_{2}{\text{A}}_{2}\\ 0 & \text{for}\;{\text{A}}_{1}{\text{A}}_{2},{\text{A}}_{2}{\text{A}}_{1}\;\text{or missing values}. \end{array}$$

The variance of the genotypic effect *g* is thus modeled as

$$Var\left(g=Zu\right)=G=ZZ^{T}\;\sigma_{u}^{2},$$ (5)

where $Z^{T}$ is the transpose of *Z* (SAS code for simulating the matrix G is provided in Supplementary Materials SAS code S1). When the model is parameterized directly in terms of genotypic effects *g* rather than marker effects *u*, the method is often referred to as GBLUP rather than RR-BLUP. Here, we use the GBLUP parameterization. A modified version of model (3) assuming that genotypic effects are independent so that $Var\left(g\right)=G=I_{n}\sigma_{u}^{2}$, where $\sigma_{u}^{2}$ is the genetic variance and $I_{n}$ is the *n*-dimensional identity matrix, was used to compute some measures of heritability.

### Real maize datasets and estimation of variance components

AgReliant Genetics provided a maize dataset with 177 genotypes and 275 markers, whereas KWS-Synbreed Project availed a larger maize dataset with 698 genotypes and 11646 markers. Marker, block, and error variance components and marker information derived from the two real maize datasets were used to simulate true breeding values and phenotypic data assuming that the genotypes are correlated (Table S2 and Table S3). To obtain smaller estimates of heritability for scenarios 4 to 6, the marker effect variances for scenarios 1 to 3 were each divided by 10. Similarly, the marker effect variances for scenarios 7 and 8 were each divided by 10 to obtain smaller estimates of heritability for scenarios 9 and 10 (Table S2 and Table S3). The variance components were estimated for the two real maize datasets and for the 1000 datasets simulated for each of the 10 scenarios using two mixed models that assume that the genotyped lines are correlated according to the linear variance–covariance structure used in the RR-BLUP model.

The AgReliant dataset contained 177 doubled haploid maize lines generated from biparental crosses. An unreplicated augmented trial design with incomplete blocks and the same common tester were used to assess the hybrid performance of testcross genotypes in terms of kernel dry weight per plot. The testcross genotypes were tested in six locations in 1 yr but not all genotypes were tested in all locations. Data from only one of the six locations were used with the RR-BLUP model to estimate the variance components used to simulate the random marker, block, and plot effects for scenarios 1 to 6 (Table S2). The selected location contained only one unreplicated trial, five blocks, two checks, and 177 lines. All the testcross genotypes and checks were genotyped and were treated alike in the RR-BLUP model. Between three and five incomplete blocks each with one row of plots were used per location. The standard varieties used to connect the different blocks enabled estimation of the inter-block variance and partitioning of the variance into block and error components. Although used to facilitate the analysis of the testcross genotypes, the standard varieties did not contribute to the prediction of *g*. All markers with more than 20% missing values, more than 5% heterozygous genotypes, or minor allele frequencies less than 2.5% were excluded from the analysis.

The KWS-Synbreed Project dataset was also taken from one of several locations contained in a larger dataset. This dataset contained 900 doubled haploid maize lines, 698 of which were genotyped but the other 202 were not. It also had six hybrid checks and five line checks. The genotypes were crossed with four testers and tested using nine trials, each of which was laid out as a 10×10 lattice square design with incomplete blocks and had two replicates. The sample size was 1800, and 38 observations lacked yield measurements. A more complete description of this dataset and how the RR-BLUP model was fitted to it has already been provided by Estaghvirou *et al.* (2013).

Because this article expands on the work of Estaghvirou *et al.* (2013), we present below a synopsis of the key statistical models and methods and refer the reader to the earlier paper for further technical details on derivations of the models.

### Simulation of datasets

#### Assumed field design, model, and outliers:

We used variance components estimated from the real maize (*Zea mays*) dataset provided by AgReliant to simulate block and plot effects (Table S2). We generated a dataset with 177 genotypes and used an α-design with two replicates to distribute these genotypes over 10 incomplete blocks per replicate, each of which had 18 plots. For this design, a suitable model must have one effect for the complete replicates and another effect for the incomplete blocks, nested within replicates. Accordingly, we simulated the field trial data using an α-design (Petersen 1994) and the model

$$y_{ijk}=µ+\gamma_{k}+b_{jk}+g_{i}+e_{ijk}$$, (6)

where $y_{ijk}$ is the yield of the *i*-th genotype in the *j*-th block nested within the *k*-th complete replicate, µ is the general effect or mean, $\gamma_{k}$ is the fixed effect of the *k*-th complete replicate, $b_{jk}$ is the random effect of the *j*-th block nested within the *k*-th complete replicate, $g_{i}$ is the random effect of the *i*-th genotype, and $e_{ijk}$ is the residual plot error associated with $y_{ijk}$. We likewise used variance components estimated from the real maize dataset with 698 genotypes provided by KWS to simulate the block and plot effects based on an α-design with two replicates and model (6) (Table S3).

### Simulation of outliers

Outliers were mimicked by adding 5-, 8-, or 10-times the SD of the residual error variance, computed from each of the two real maize datasets and used for the simulations, to a single randomly chosen observation in each simulated dataset. All the simulated datasets based on variance components estimated from the real maize datasets used the three multiples of the exact same SD: 6.977 for the small and 7.340 for the large datasets. The correlation between the predicted and the true breeding values, predictive accuracy, was estimated by each of four different indirect (methods 1 to 4) and three direct (methods 5 to 7) methods for each of the simulated datasets with and without outliers. A total of 10 simulation scenarios were defined by varying the configuration of the number of genotypes, size of marker effect variance, and multiples of the two SDs used in simulating the error variances (Table 1). The three outliers were carefully chosen to represent the three scenarios likely to be encountered in practice. The smallest outlier was chosen so that it would be difficult to detect using standard outlier diagnostic tools such as plots of Studentized residuals against the predicted mean and Q-Q plots (Figure S1 and Figure S2). By contrast, the largest outlier was easily detectable using these diagnostic tools (Figure S3 and Figure S4), whereas the difficulty with which the middling outlier would be detected was intermediate between the other two (Figure S5).

**Table 1:** The 10 simulated scenarios defined by configurations of the number of genotypes, the number of markers, the size of the marker effect variance, and the size of outliers

Scenario	Genotypes	Markers	Marker Variance	Error Variance (σ)	Outliers
1	177	275	0.2019	6.977	5 × 6.977
2	177	275	0.2019	6.977	8 × 6.977
3	177	275	0.2019	6.977	10 × 6.977
4	177	275	0.2019/10	6.977	5 × 6.977
5	177	275	0.2019/10	6.977	8 × 6.977
6	177	275	0.2019/10	6.977	10 × 6.977
7	698	11,646	0.005892	7.340	5 × 7.340
8	698	11,646	0.005892	7.340	10 × 7.340
9	698	11,646	0.005892/10	7.340	5 × 7.340
10	698	11,646	0.005892/10	7.340	10 × 7.340

Data expressed as multiples of the standard deviations of the error variances.

### Estimating predictive accuracy

Predictive accuracy $r_{g,\hat{g}}$ was estimated either indirectly (methods 1 to 4) or directly (methods 5 to 7). Indirect estimation of predictive accuracy involved dividing predictive ability, the correlation between the estimated breeding values, and the observed phenotypic values $\left(r_{\hat{g},p}\right)$, by the square root of heritability $H^{2}$ $\left(r_{\hat{g},p}/H\right)$ following the work of Legarra *et al.* (2008). By contrast, direct estimation of predictive accuracy does not involve the use of heritability. Because methods 1 to 4 use cross-validation, predictive accuracy was calculated for each of 15 three-fold cross-validation replicates derived for each simulated dataset. A common estimate of predictive ability used with methods 1 to 4 is given by:

$$r_{\hat{g},p}=\frac{S_{\hat{g,}p}}{\sqrt{S_{\hat{g}}^{2}S_{p}^{2}}}$$, (7)

where $s_{\hat{g,}p}$, is the covariance between the predicted breeding values and the observed phenotypic values, $s_{\hat{g}}^{2}$, and $s_{p}^{2}$ are defined as in equation (1).

Two important assumptions are implicit in equation (7). The first is that $s_{\hat{g,}p}=s_{g,\hat{g}}$ (Dekkers 2007), which is equivalent to assuming that the unobserved genotypic effect *g* is not correlated with the environmental components in *p*. Accordingly, on substituting $s_{g,\hat{g}}$ in equation (1) with $s_{\hat{g,}p}$ (Dekkers 2007), we obtain

$$r_{g,\hat{g}}=\frac{s_{\hat{g,}p}}{\sqrt{s_{\hat{g}}^{2}s_{g}^{2}}},$$ (8)

where all the terms are defined as in equations (1) and (7). The second crucial assumption implicit in equation (7) is that $s_{g}^{2}=H^{2}s_{p}^{2}$, from which we deduce that

$$r_{g,\hat{g}}=\frac{s_{\hat{g,}p}}{\sqrt{s_{\hat{g}}^{2}s_{g}^{2}}}=\frac{s_{\hat{g},p}}{H\sqrt{s_{\hat{g}}^{2}s_{p}^{2}}}=\frac{r_{\hat{g},p}}{H}$$. (9)

From equation (9), all that remains to compute $r_{g,\hat{g}}$ is to specify how to compute heritability $H^{2}$. To do this, we recall that the definition of $\hat{g}$ used in $r_{g,\hat{g}}$ requires a marker-based model for *g*. It is thus logical to use the same model to define heritability $H^{2}$. However, working with the new definition is complicated by the fact that the true model is not known and must be approximated by suitable methods for genomic prediction. One way to overcome this complication is to estimate predictive accuracy using the same model used to define predictive accuracy, provided the model is close to the model for some genomic prediction method. Such a model should be chosen judiciously, however, because some methods for genomic prediction, for instance, in the machine learning field, lack explicit underlying models.

A precise definition of heritability $H^{2}$ is thus impeded by the difficulty inherent in choosing a suitable model for heritability. Because of this intrinsic difficulty, any estimate of predictive accuracy should be regarded as furnishing only an approximation of precision. Because the model underlying ridge regression BLUP is the most widely used method for genomic prediction, possesses several attractive properties, uses a specific mixed model, and allows an estimate of $H^{2}$to be obtained in several different ways (Piepho and Möhring 2007), we use it to define heritability $H^{2}$.

### Methods for estimating heritability and predictive accuracy

We used five approaches (methods 1 to 5) to estimate heritability for the datasets with and without outliers. The first method is commonly used by plant breeders (Albrecht *et al.* 2011) to estimate heritability. The second and the third methods are modifications of the *ad hoc* measure using BLUE and BLUP (Piepho and Möhring 2007). The fourth method used to estimate heritability was proposed by Estaghvirou *et al.* (2013). This method uses the ratio of the expected value of the genetic variance to the expected value of the phenotypic variance. The fifth method was the second method proposed by Estaghvirou *et al.* (2013) to estimate heritability without cross-validation based on ideas similar to those used in computing the *ad hoc* measures of heritability $H^{2}$. Methods 1 to 3 assume the genotypes are independent, whereas methods 4 and 5 assume that the genotypes are correlated according to the linear variance–covariance structure assumed to underlie the RR-BLUP model. The quantity $\left(r_{\hat{g},p}/H\right)$ was used to estimate predictive accuracy, where *H* is estimated using each of the first four approaches (methods 1 to 4) only and $r_{\hat{g},p}$ is the Pearson product-moment correlation between the estimated breeding values and the simulated phenotypes. This is because, despite estimating heritability, method 5 computes predictive accuracy directly, as do methods 6 and 7. Methods 5 to 7 were thus used to estimate predictive accuracy for the datasets with and without outliers directly without first dividing predictive ability by the square root of heritability. The three direct approaches assume that the effects of genotypes are correlated according to the model underlying the RR-BLUP.

We now present the essential details of each method in what follows.

#### Method 1:

This method first computes heritability using the relation (Legarra *et al.* 2008)

$$H_{m_{1}}^{2}=\frac{\sigma_{g}^{2}}{\sigma_{g}^{2}+\sigma_{e}^{2}/r},$$ (10)

where $\sigma_{g}^{2}$ is the genetic variance, *r* is the number of replicates, and $\sigma_{e}^{2}$ is the variance of plot error. Plant breeders routinely use this estimator with randomized complete block designs as an *ad hoc* approximation with incomplete block designs. However, the method is not appropriate for spatially correlated data (Gonçalves *et al.* 2013).

#### Method 2:

This method uses an estimator based on the BLUEs and first computes heritability using the relation (Piepho and Möhring 2007)

$$H_{m_{2}}^{2}=\frac{\sigma_{g}^{2}}{\sigma_{g}^{2}+\bar{\upsilon }/2},$$ (11)

where $\bar{\upsilon }$ is the mean variance of a difference of two adjusted genotypic means (BLUE) and $\sigma_{g}^{2}$ is the genetic variance estimated using equation (6), assuming uncorrelated genotypic effects.

#### Method 3:

This method computes an *ad hoc* measure of heritability based on BLUP and assumes that genotypic effects are not correlated (Cullis *et al.* 2006)

$$H_{m_{3}}^{2}=1-\frac{{\bar{\upsilon }}_{BLUP}}{2\sigma_{g}^{2}},$$ (12)

where ${\bar{\upsilon }}_{BLUP}$ is the mean variance of a difference of the BLUP of two genotypic effects ${\hat{g}}_{i}$. The BLUP of *i* was used as the phenotypic data in the mixed model for method 3.

#### Method 4:

This method defines heritability as the expected genetic sample variance $s_{g}^{2}$ divided by the expected phenotypic sample variance $s_{p}^{2}$

$$H_{m_{4}}^{2}=\frac{E\left(s_{g}^{2}\right)}{E\left(s_{p}^{2}\right)}$$, (13)

where

$$E\left(s_{g}^{2}\right)=trace\left(P_{u}G\right)\;\text{and}\;E\left(s_{p}^{2}\right)=trace\left(VP_{u}\right)=E\left(s_{g}^{2}\right)+trace\left(RP_{u}\right)$$ (14)

with $G=ZZ^{T}\sigma_{u}^{2}$ and *R* being the variance–covariance matrices of genotypes and errors, respectively, $P_{u}=\frac{1}{n-1}\left(I_{n}-\frac{1}{n}J_{n}\right)$, with $I_{n}$ denoting an *n*-dimensional identity matrix and $J_{n}=n\times n$ is a square matrix of ones. Heritability in equation (13) is estimated by plugging in estimates of *G* and *R*.

#### Method 5:

This method directly defines both the predictive accuracy and heritability as

$$E\left(r_{g,\hat{g}}\right)\approx H_{m_{5}}=\frac{trace\left(P_{u}CG\right)}{\sqrt{trace\left(P_{u}G\right)trace\left(C^{T}P_{u}CV\right)}}$$, (15)

where $G=ZZ^{T}\sigma_{u}^{2}$ and *R* are the variance–covariance matrices of genotypes and errors. Var(p)=V=G+R is the variance–covariance matrix of the phenotypes, $P_{u}$ is defined as above, and $C=GV^{-1}Q$ with $Q=I-1{\left(1^{T}V^{-1}1\right)}^{-1}1^{T}V^{-1}$, where $V^{-1}$ is the inverse of the variance–covariance matrix of the phenotypes and 1 is a vector of ones.

#### Method 6:

This method directly estimates predictive accuracy using the relation

$$r_{g,\hat{g,}m_{6}}=\frac{s_{\hat{g},p}}{\sqrt{s_{\hat{g}}^{2}\;E\left(s_{g}^{2}\right)}}$$, (16)

where $s_{\hat{g}}^{2}$ is the sample variance of the predicted breeding values $\left(\hat{g}\right)$, $s_{\hat{g},p}$ is the sample covariance between the predicted breeding values $\left(\hat{g}\right)$, and the phenotypic values *p* and $E\left(s_{g}^{2}\right)$ is estimated using equation 14.

#### Method 7:

This method is commonly used in animal breeding to directly compute predictive accuracy from the mixed model equations based on reliability $\rho_{i}^{2}$ as (Mrode and Thompson 2005; Henderson 1975; Van Raden 2008):

$$\rho_{i}^{2}=\frac{{\left(cov\left(g_{i},{\hat{g}}_{i}\right)\right)}^{2}}{var\left(g_{i}\right)\;var\left({\hat{g}}_{i}\right)},$$ (17)

where var(g) and $var\left(\hat{g}\right)$ are the variances of the true and predicted breeding values and $cov\left(g,\hat{g}\right)$ is the covariance between the true and predicted breeding values. The reliability of all the genotypes in each dataset is then estimated by:

$${\hat{\rho }}_{m_{7}}^{2}=\frac{1}{n}\sum_{i=1}^{n}{\hat{\rho }}_{i}^{2}$$ (18)

from which the estimated predictive accuracy is computed as the square root of reliability ${\hat{\rho }}_{m_{7}}^{2}$, where *n* is the total number of genotypes in the dataset. The practical details of how the statistics involving the true breeding values are actually estimated from the mixed model, despite the fact that the true breeding values are unknown, are omitted here because they are provided by Estaghvirou *et al.* (2013).

### Cross-validation

The correlation between the predicted breeding values and the observed phenotypic values$\left(r_{\hat{g,}p}\right)$, called predictive ability, was estimated for methods 1 to 4 and 6 using a threefold cross-validation (CV) and was used to estimate predictive accuracy. The choice of the threefold cross-validation procedure was dictated by the small number of genotypes (177) in the AgReliant dataset. The dataset with the adjusted means for the testcross genotypes was split into three random subsamples, one of which was held out in turn as a validation set. The other two subsamples were amalgamated into a single training set. The cross-validation procedure was replicated five times to yield 15 replicate datasets. A ridge regression model (6 and 7) was fitted to each of the 15 replicate validation and training sets. Predictive ability was then computed for all the genotypes. This procedure was repeated for each of the 1000 datasets simulated for each of the 10 scenarios. Predictive accuracy, the correlation between the predicted and the true breeding values $r_{g,\hat{g}}$, was estimated by the ratio of predictive ability $s_{\hat{g,}p}$ to the square root of heritability for each of the indirect methods using datasets with and without outliers. Moreover, we directly estimated predictive accuracy using methods 5, 6, and 7 for the datasets with and without outliers as detailed below. The estimated predictive accuracy for each method for the datasets with outliers was compared with the estimates for the corresponding datasets without outliers. Furthermore, we compared all seven methods among themselves using the datasets with outliers.

### Evaluation of the simulated data

The two-stage analysis and the preceding methods were used to estimate the predictive accuracy for the datasets with and without outliers. We also estimated heritability using each of the methods 1 to 5. The adjusted means (p) of the genotypes computed from the simulated data at the first stage were used as the phenotypic data in the second stage of the cross-validatory analyses. Equation 11 was used to compute an *ad hoc* measure of heritability from the variance–covariance matrix of the adjusted means.

### Comparing heritabilities

We estimated heritability for the simulated datasets with and without outliers for each of the 10 scenarios using each of the four different indirect methods (methods 1 to 4) and method 5. We then used the mean deviation (MD) and its SE to quantify the deviations in the estimated heritability computed for the *j*-th dataset with outliers ${\hat{r}}_{g,\hat{g},o,j}^{2}$ from the corresponding simulated true heritability, regarded as the benchmark, $r_{g,\hat{g},j}^{2}$:

$$MD_{H2out}=\sum_{j=1}^{1000}\frac{\left({\hat{r}}_{g,\hat{g},o,j}^{2}-r_{g,\hat{g},j}^{2}\right)}{1000},$$ (19)

Similarly, we used MD and its SE to quantify the deviation of the estimated heritability for the *j*-th dataset without outliers ${\hat{r}}_{g,\hat{g},j}^{2}$ from the corresponding simulated true heritability:

$$MD_{H2norm}=\sum_{j=1}^{1000}\frac{\left({\hat{r}}_{g,\hat{g},j}^{2}-r_{g,\hat{g},j}^{2}\right)}{1000},$$ (20)

Further, we used MD to compare the estimated heritability between pairs of all five methods.

Finally, we computed descriptive statistics for the deviations in the estimated heritability for the datasets with outliers from the simulated true heritability as the benchmark and compared them with the corresponding deviation of the heritability estimates for the datasets without outliers from the simulated true heritability.

### Comparing predictive accuracies

The simulated true predictive accuracy for the *j*-th dataset $r_{g,\hat{g},j}$ was computed as the correlation between the simulated true and the predicted breeding values for each of the 1000 datasets simulated for each of the 10 configurations of number of genotypes, marker effect variances, and outliers (Table S4). Each of the seven methods was then used to estimate predictive accuracy as the correlation between the simulated true and the predicted breeding values for the *j*-th dataset simulated with $\left({\hat{r}}_{g,\hat{g},o,j}\right)$ and without $\left({\hat{r}}_{g,\hat{g},j}\right)$ outliers. The MD of the estimated predictive accuracy for the *j*-th dataset with outliers from the corresponding simulated predictive accuracy was then computed as:

$$MD_{Accout}=\sum_{j=1}^{1000}\frac{\left({\hat{r}}_{g,\hat{g},o,j}-r_{g,\hat{g},j}\right)}{1000},.$$ (21)

In the same vein, the MD for the estimated predictive accuracy for the *j*-th dataset without outliers from that for the corresponding simulated predictive accuracy was calculated as:

$$MD_{Accnorm}=\sum_{j=1}^{1000}\frac{\left({\hat{r}}_{g,\hat{g},j}-r_{g,\hat{g},j}\right)}{1000},$$ (22)

Moreover, for each method and scenario combination, the deviation of the estimated predictive accuracy for the datasets with outliers from the simulated true predictive accuracy was compared with the deviation of the estimated predictive accuracy for the datasets without outliers from the simulated predictive accuracy as the benchmark. We also compared estimates of MD between pairs of the seven methods both within and between scenarios.

**Figure 1:**
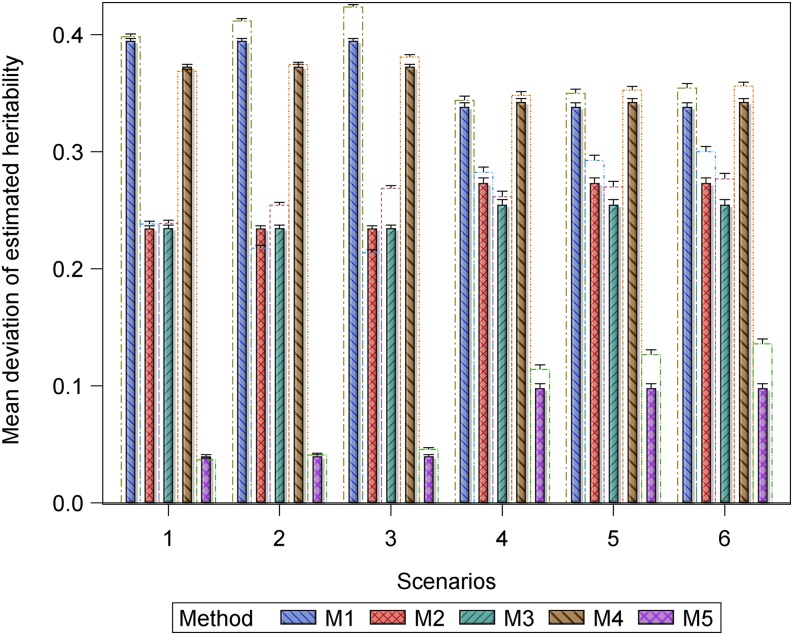
Bar plots for the mean deviation (MD) in the estimated heritability for the datasets with ${\hat{r}}_{g,\hat{g},o}^{2}$ (empty bars) and without ${\hat{r}}_{g,\hat{g}}^{2}$ (filled and hatched bars) outliers, regarded as the benchmark, from the simulated true heritability $r_{g,\hat{g}}^{2}$ for each of the five methods for estimating heritability in scenarios 1 to 6. Scenarios 1 to 3 are based on the same 1000 datasets simulated assuming 177 genotypes and a marker effect variance of 0.2019 and scenarios 4 to 6 are based on the same 1000 datasets simulated assuming 177 genotypes and a marker effect variance of 0.2019/10.

**Figure 2:**
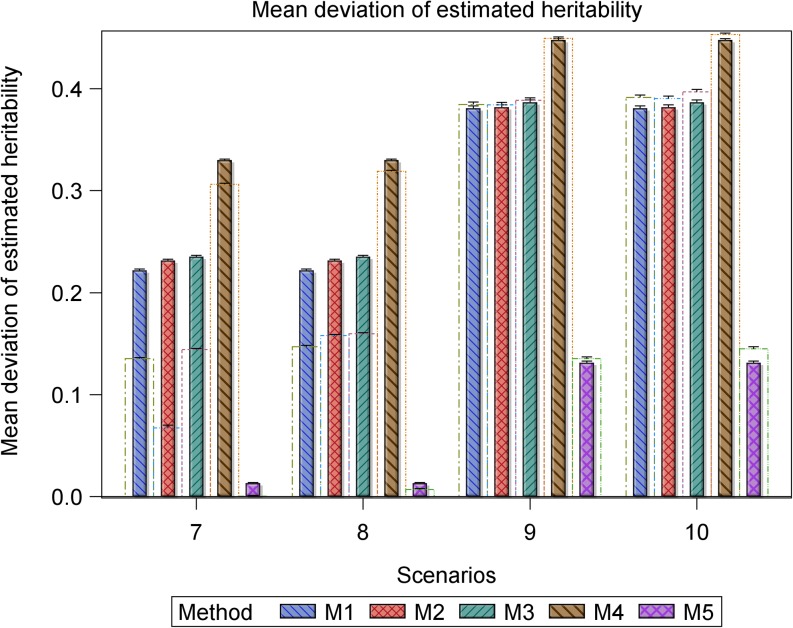
Bar plots for the mean deviation (MD) in the estimated heritability for the datasets with ${\hat{r}}_{g,\hat{g},o}^{2}$ (empty bars) and without ${\hat{r}}_{g,\hat{g}}^{2}$ (filled and hatched bars) outliers, regarded as the benchmark, from the simulated true heritability $r_{g,\hat{g}}^{2}$ for each of the five methods for estimating heritability in scenarios 7 to 10. Scenarios 7 and 8 are based on the same 1000 datasets simulated assuming 698 genotypes and a marker effect variance of 0.005892, whereas scenarios 9 and 10 are based on the same 1000 datasets simulated assuming 698 genotypes and a marker effect variance of 0.005892/10.

Also, we calculated and compared descriptive summaries for the deviations of the estimated predictive accuracies for the datasets with outliers from the benchmark accuracies with the summaries for the deviations for the datasets without outliers from the benchmark accuracies. Finally, we used frequency histograms, box whisker plots, and bar charts to compare the deviation and mean deviation estimates between methods within and between scenarios (Figure 1, Figure 2, Figure 4, and Figure 5, Figure S6 and Figure S7).

For each of the 10 scenarios we compared the difference in the deviations of the estimated predictive accuracies for the datasets with and without outliers from the simulated predictive accuracy between all pairs of the seven methods using Pearson correlations and *t*-tests (α=5%) adjusted for multiplicity using simulation adjustment. The *t*-tests were based on a mixed model with fixed effects for method and scenario and their interaction and a random effect for simulation replicates nested within scenarios (Piepho *et al.* 2012b).

## Results

### Heritability

Only five (methods 1 to 5) of the seven methods estimate heritability. For the small datasets (*n* = 177), the accuracy of the estimated heritability decreased as the magnitude of the outliers increased or as the genetic variance decreased for all the five methods based on the deviation of the simulated true heritability from the heritability estimated using datasets with or without outliers (Table S4 and Table S5, Figure 1 and Figure 2). Across all the scenarios based on the same datasets, outliers evidently reduced the accuracy with which heritability was estimated (Table S4 and Table S5, Figure 1 and Figure 2). Moreover, reducing the genetic variance by a factor of 10 in the presence of outliers reduced the accuracy of the estimated heritability even more in scenarios 4 to 6 relative to scenarios 1 to 3, whereas increasing the size of the outliers markedly degraded accuracy in scenarios 9 to 10 relative to scenarios 7 and 8. For the large datasets, this pattern was less clear-cut. Of the seven methods, outliers had the least influence on method 5 in all the 10 scenarios. Consequently, method 5 was the most resistant to increasing the magnitude of outliers or additionally decreasing the genetic variance, followed by methods 2, 3, 4, and 1, in decreasing order (Table S4 and Table S5, Figure 1 and Figure 2).

### Predictive accuracy

**Figure 3:**
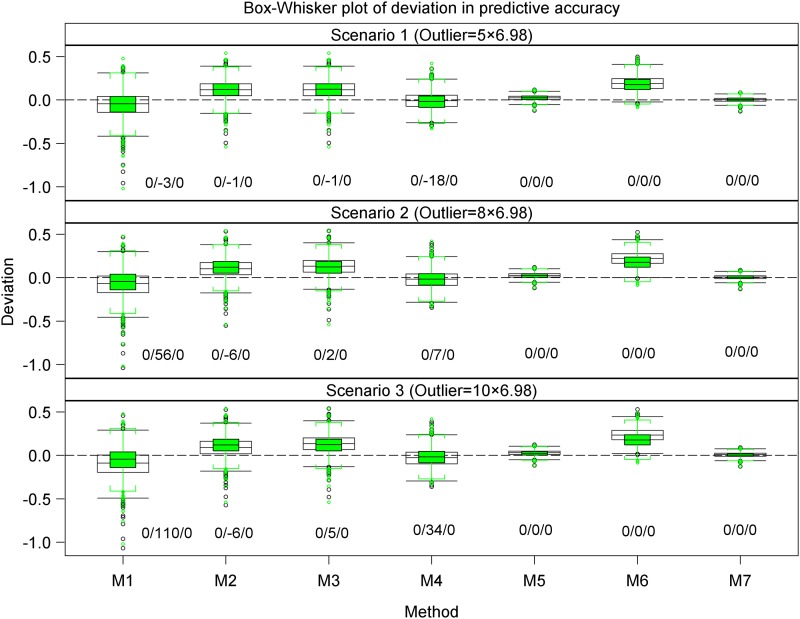
Box whisker plots of the deviations in the estimated predictive accuracy for the datasets with ${\hat{r}}_{g,\hat{g},o}$(empty box and whiskers capped with brackets) and without ${\hat{r}}_{g,\hat{g}}$ (green boxes) outliers, regarded as the benchmark, from the simulated true predictive accuracy $r_{g,\hat{g}}$ for each of the seven methods in scenarios 1 to 3. All the scenarios are based on the same 1000 datasets simulated assuming 177 genotypes and a marker effect variance of 0.2019. a/b/c denotes the difference in the number of datasets with outliers for each scenario, out of a possible total of 1000, that showed undershooting, overshooting or did not converge relative to the numbers for the corresponding datasets without outliers.

**Figure 4:**
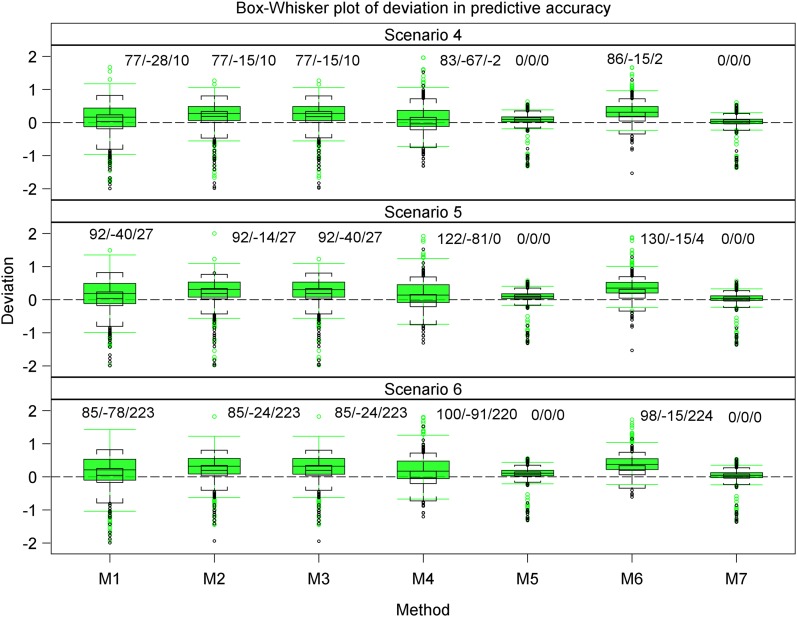
Box whisker plots of the deviations in the estimated predictive accuracy for the datasets with ${\hat{r}}_{g,\hat{g},o}$ (empty box and whiskers capped with brackets) and without ${\hat{r}}_{g,\hat{g}}$ (green boxes) outliers, regarded as the benchmark, from the simulated true predictive accuracy $r_{g,\hat{g}}$ for each of the seven methods in Scenarios 4-6. All the scenarios are based on the same 1000 datasets simulated assuming 177 genotypes and a marker effect variance of 0.2019/10. Values presented as “number/number/number” denote the difference in the number of datasets with outliers for each scenario, out of a possible total of 1000, that showed undershooting, overshooting, or did not converge relative to the numbers for the corresponding datasets without outliers.

**Figure 5:**
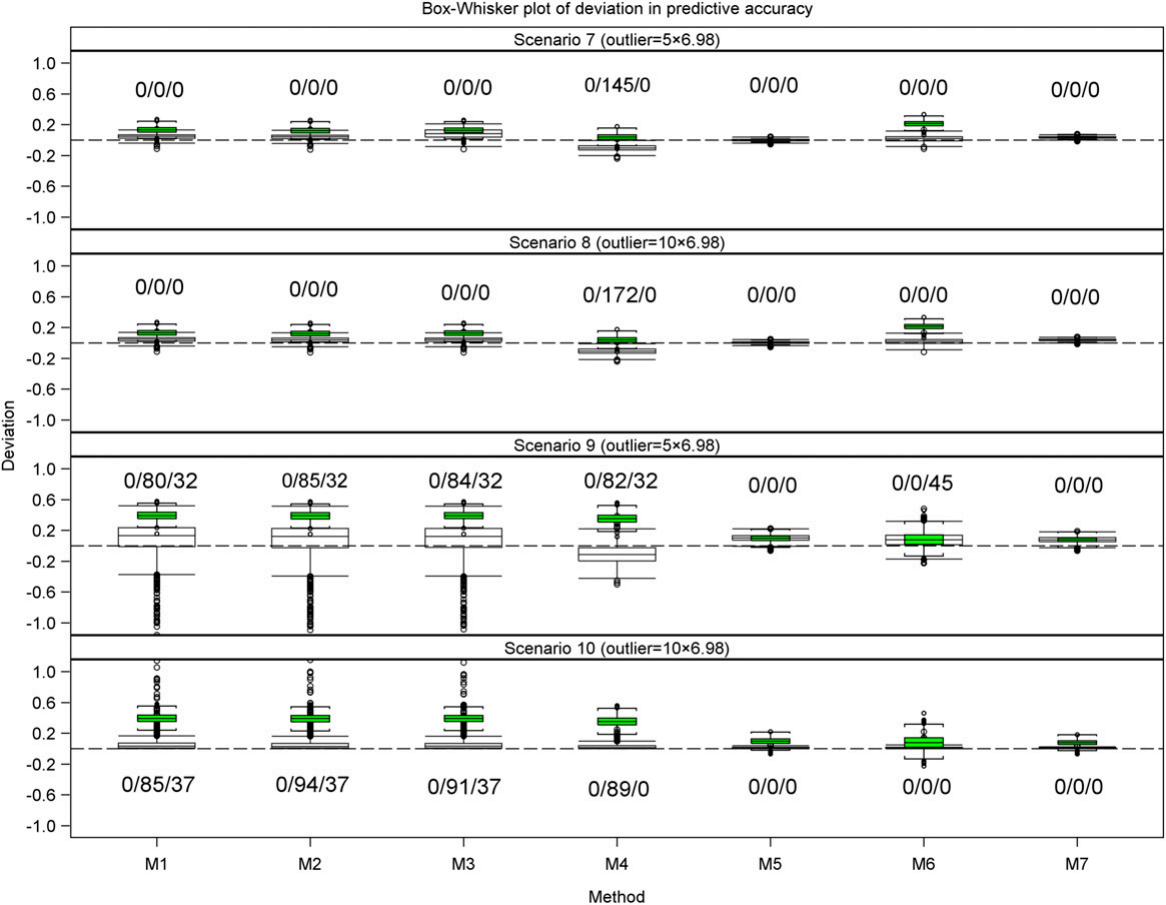
Box whisker plots of the deviations in the estimated predictive accuracy for the datasets with ${\hat{r}}_{g,\hat{g},o}$ (empty box and whiskers capped with brackets) and without ${\hat{r}}_{g,\hat{g}}$ (green boxes) outliers, regarded as the benchmark, from the simulated true predictive accuracy $r_{g,\hat{g}}$ for each of the seven methods in scenarios 7 to 10. All the scenarios are based on the same 1000 datasets simulated assuming 698 genotypes and a marker variance of 0.005892 for scenarios 7 and 8 and 0.005892/10 for scenarios 9 and 10. Values presented as “number/number/number” denote the difference in the number of datasets with outliers for each scenario, out of a possible total of 1000, that showed undershooting, overshooting, or did not converge relative to the numbers for the corresponding datasets without outliers.

**Figure 6:**
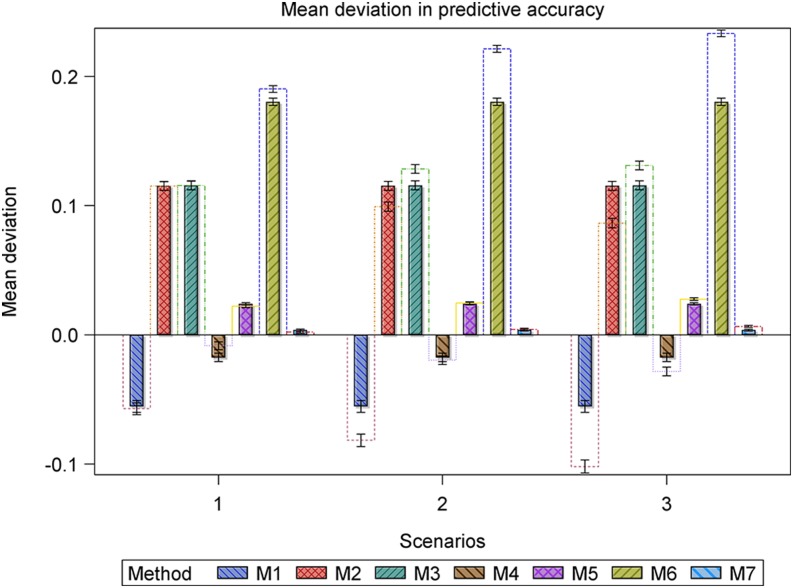
Bar plots for the mean deviation (MD) of the estimated accuracy for the datasets with ${\hat{r}}_{g,\hat{g},o}$ (empty bars) and without ${\hat{r}}_{g,\hat{g}}$ (filled and hatched bars) outliers, regarded as the benchmark, from the simulated true predictive accuracy $r_{g,\hat{g}}$ for each of the seven methods in scenarios 1 to 3. All the scenarios are based on the same 1000 datasets simulated assuming 177 genotypes and a marker effect variance of 0.2019.

**Figure 7:**
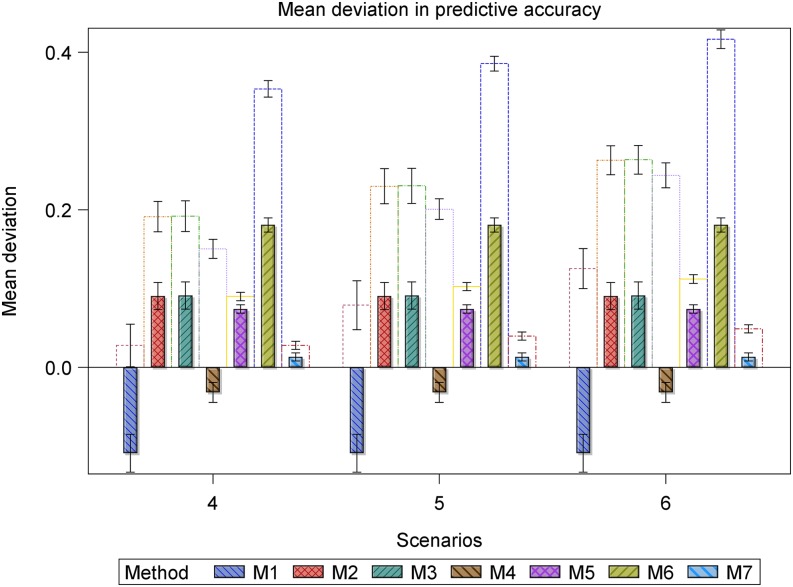
Bar plots for the mean deviation (MD) of the accuracy for the datasets with ${\hat{r}}_{g,\hat{g},o}$ (empty bars) and without ${\hat{r}}_{g,\hat{g}}$ (filled and hatched bars) outliers, regarded as the benchmark, from the simulated true predictive accuracy $r_{g,\hat{g}}$ for each of the seven methods in scenarios 4 to 6. All the scenarios are based on the same 1000 datasets simulated assuming 177 genotypes and a marker effect variance of 0.2019/10.

**Figure 8:**
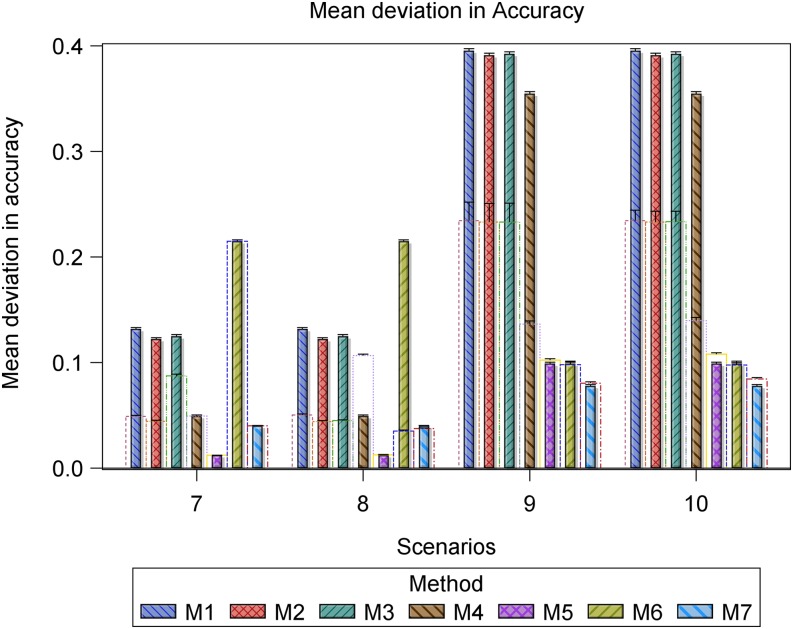
Bar plots for the mean deviation (MD) of the accuracy for the datasets with ${\hat{r}}_{g,\hat{g},o}$ (empty bars) and without ${\hat{r}}_{g,\hat{g}}$ (filled and hatched bars) outliers, regarded as the benchmark, from the simulated true predictive accuracy $r_{g,\hat{g}}$ for each of the seven methods in scenarios 7 to 10. All the scenarios are based on the same 1000 datasets simulated assuming 698 genotypes and a marker effect variance of 0.005892 for scenarios 7 and 8 and 0.005892/10 for scenarios 9 and 10.

Overall, outliers adversely affected the estimated predictive accuracy for all seven methods but the severity of this effect varied across methods and scenarios. Nevertheless, some methods (*e.g.*, 2 and 3 or 5 and 7) responded similarly to outliers across all 10 scenarios. Outliers had the least influence on the estimated predictive accuracies for methods 5 and 7 across all 10 scenarios (Table S5, Figure 3, Figure 4, Figure 5, Figure 6, Figure 7, and Figure 8, Figure S6, Figure S7, and Figure S8). Methods 5 and 7 were therefore the most robust: to outliers and also gave the best estimates for predictive accuracy. For the five methods more adversely affected by outliers, the specific types of adverse effects of outliers on the estimated predictive accuracy were manifold and varied by both sample size (number of genotypes) and the genetic variance. More precisely, outliers degraded estimation accuracy the most for the small datasets with reduced genetic variance. Outliers were associated with a greater tendency to underestimate predictive accuracy for the small datasets in scenarios 1 to 6 (Table S5, Figure 3, Figure 4, Figure 6, and Figure 7, Figure S6 and Figure S7), to overestimate predictive accuracy for the large datasets in scenarios 7 to 10 (Table S5, Figure 5 and Figure 8, Figure S8), and a greater likelihood of the mixed models to fail to converge. Overestimation was more serious for methods 1 to 4 than 5 and 7 in scenarios 7 to 10. The degree of underestimation or overestimation increased with increasing magnitude of the outliers.

We now elaborate on the specifics of the adverse effects of outliers on accuracy estimation by method and scenario. Even though all seven methods exhibited varying degrees of robustness to outliers, clear differences were often discernible in their performances. In general, the modification of the influence of outliers on accuracy estimation of the seven methods by the genetic variance became more severe when the genetic variance was reduced. For example, dividing the genetic variance by 10 but leaving all the other variance components intact was associated with a marked increase in the frequency of undershooting (estimates of predictive accuracy less than 0), a decrease in the frequency of overshooting (estimates of predictive accuracy more than 1), and increase in the frequency of datasets for which the mixed models failed to iterate to convergence (Figure 4, Figure 5, and Figure 8, Figure S7 and Figure S8). The degree to which genetic variance modified the influence of outliers varied, in turn, with sample size (number of genotypes), such that outliers had the greatest adverse effect on the estimated predictive accuracy for the small datasets (*n* = 177 genotypes) with reduced genetic variance.

The deviations in the estimated predictive accuracy from the simulated true accuracy with and without (benchmark) outliers showed that outliers had the smallest, albeit similar, effects on methods 5 and 7 but were associated with overestimation of accuracy for all methods except 5 and 7 (Table S5, Figure 3, Figure 4, and Figure 5, Figure S7). Furthermore, outliers were also associated with underestimation of the variance of predictive accuracy across replicate datasets for all the methods in scenarios 1 to 6 (Table S5, Figure 3 and Figure 4). Even so, outliers did not alter the relative performances of the seven methods established in the absence of outliers.

Across all the scenarios, methods 5 and 7 produced the most accurate predictions in scenarios 1 to 6. But a comparison of scenarios 1 to 3 with 4 to 6, all of which are based on datasets with the same sample size but different genetic variances, makes it apparent that reducing the genetic variance markedly reduces predictive accuracy, even in the absence of outliers. However, reducing the genetic variance does not alter the relative performances of the seven methods or the general tendency of the estimated accuracy to decrease with increasing magnitude of outliers in scenarios 1 to 3 compared with scenarios 4 to 6. Instead, it greatly increases convergence problems, most critically for methods 1 to 3 (Figure 4 and Figure 5), and the variance of the benchmark predictive accuracy for all seven methods, particularly for methods 1 to 4 and 6 (Table S5, Figure 4, Figure 5, and Figure 6). Methods 5 and 7 still gave the most precise accuracy estimates even when the genetic variance was reduced.

For all seven methods, outliers did not cause any noteworthy change in the frequency of undershooting in the estimated predictive accuracy for either the small (scenarios 1 to 3) or the large (scenarios 7 and 8) simulated datasets with large variances (Figure 3 and Figure 5). But when the genetic variances were reduced by a factor of 10, the cases of undershooting in the estimated predictive accuracy became clearly more pronounced for the small simulated datasets with outliers for all the methods except 5 and 7 in scenarios 4 to 6 (Figure 4 and Figure 5). The extent of the increase in cases of undershooting for methods 1 to 4 and 6 in scenarios 4 to 6 also tended to increase with increasing size of the outliers (Figure 4 and Figure 5). The same 10-fold reduction in genetic variance similarly increased convergence problems and the frequency of overshooting in the estimated predictive accuracy for the simulated large datasets with outliers for all the seven methods, especially for methods 1 to 4 in scenarios 9 and 10 (Figure 5).

Outliers did not materially change the number of datasets for which the estimated predictive accuracy exceeded 1 (*i.e.*, overshooting) for methods 2, 3, 5, 6, and 7, but was associated with either increased (method 1) or decreased (method 4) frequency of overshooting in scenarios 1 to 3 (Figure 3). The magnitude of change in the frequency of overshooting for methods 1 and 4 increased with increasing size of the outliers. The frequency of overshooting decreased for the small datasets with outliers compared to the same size datasets without outliers for methods 1 to 4 and 6, but it did not change for methods 5 and 7 in scenarios 4 to 6 (Figure 4). Also, the degree of the decrease in the frequency of overshooting increased with increasing size of the outliers. By contrast, for the large datasets the frequency of overshooting increased only for method 4 in scenarios 7 and 8 and for methods 1 to 4 in scenarios 9 and 10 (Figure 5). The increase in the frequency of overshooting tended to increase with increasing size of the outliers (Figure 4 and Figure 5).

For all seven methods except 5 and 7, both of which were the most resistant to outliers, outliers were also associated with a greater tendency for the mixed models to fail to iterate to convergence, zero, or very small estimated heritability for both the small and large datasets with small genetic variances. Thus, for scenarios 4 to 6 and 9 to 10 based on the datasets with reduced genetic variances, there was a clear increase in the number of datasets for which the mixed models failed to converge as the magnitude of the outliers increased (Figure 4 and Figure 5). This increase was more pronounced for the small than for the large datasets (scenarios 4 to 6) (Figure 4).

However, all the methods that use cross-validation (methods 1 to 4 in scenarios 7 and 8 in Figure 2 and methods 1 to 4 and 6 in scenarios 7 to 10 in Figure 8) show a somewhat anomalous and counterintuitive pattern that deviates from the expected pattern and seems to suggest, quite intriguingly, that outliers enhance prediction. This unexpected behavior can be explained by two different biases of opposing sign. Thus, for example, if a method shows a large negative bias in the estimation of predictive accuracy, whereas the additional bias caused by outliers is positive in sign, the two biases may partly annihilate each other, leading to the somewhat unexpected behavior we observed. Nevertheless, we emphasize that this behavior was not found for the two best performing methods 5 and 7.

The pairwise comparisons using *t*-tests reinforced the preceding patterns, including the observation that methods 5 and 7 gave similar and the most accurate estimates of predictive accuracy (Table S5). These tests demonstrated clear variation among methods in terms of their performances. The performances of methods 5 and 7 were similar and outstanding in several important respects. First, all their estimated predictive accuracies ranged between 0 and 1 in all 10 scenarios (Figure 3, Figure 4, and Figure 5). Second, the mixed models for methods 5 and 7 converged for all 1000 datasets simulated for each of the 10 scenarios (Figure 3, Figure 4, and Figure 5). In contrast to methods 5 and 7, the remaining methods were much less consistent in their sensitivity to outliers across scenarios. Methods 2 and 3 also responded similarly to outliers as indicated by the very high similarities of their estimated predictive accuracies across all the 10 scenarios (Table S5). The inconsistency in response to outliers was apparent in an increased likelihood of overestimating accuracy when outliers are present than when they are not in scenarios 1 to 6 (Figure 3 and Figure 4). It was also manifested in a higher frequency of failure of the mixed models to converge in scenarios 4 to 6 than in scenarios 1 to 3. We recall that both sets of scenarios are based on datasets with the exact same sample size and outliers. The only important difference between them is that the datasets in scenarios 4 to 6 have 10-times smaller genetic variances than those in scenarios 1 to 3 (Figure 1 and Figure 2). The relatively close match between the estimated and the benchmark accuracy for all seven methods in scenarios 1 to 3 (Figure 3 and Figure 6, Figure S6) but striking overestimation of the benchmark accuracy by all methods but methods 5 and 7 in scenarios 4 to 6 (Table S5, Figure 4 and Figure 7, Figure S7) can thus be attributed to the reduced genetic variance. Underestimation of the benchmark predictive accuracy was another consequence of outliers that was common for all seven methods but methods 5 and 7 in scenarios 7 to 10 (Table S5, Figure 5 and Figure 8). Three further shortcomings were apparent for all the methods but methods 5 and 7 in scenarios 4 to 6 and 9 to 10. The first was that predictive accuracy could not be computed because the estimated heritability was zero, in particular for methods 1 to 3 (*n* = 193 datasets) in scenario 6. The other deficiency was that the estimated genetic variance was sometimes zero [*e.g.*, for methods 1 to 3 (*n* = 30) in scenario 6], thereby precluding the computation of predictive ability. Finally, the mixed models sometimes failed to converge (*n* = 219), so that the results required by methods 1 to 4 and 6 to compute predictive ability in scenario 6 were unavailable in these cases.

The *t*-tests showed that the seven methods clustered into two fairly distinct groups, with each consisting of methods with comparable performances in terms of their responses to outliers in each of the 10 scenarios. The first cluster consisted of methods 1 to 4 and 6 and the other consisted of methods 5 and 7 (Table S5, Figure 3, Figure 4, Figure 5, Figure 6, Figure 7, and Figure 8). The estimated predictive accuracies for each of the methods were often high and positive, but some methods (mainly 5 and 7) tended to produce high predictive accuracies when others (*e.g.*, methods 1 and 4) produce low accuracies for the same datasets and scenarios (results not shown).

## Discussion

Outliers have long been known to present serious complications to regression problems (Cook and Weisberg 1982; Belsley *et al.* 2005; Schabenberger 2004); therefore, they can be expected *a priori* to similarly do so to genomic prediction problems. However, to our knowledge, the influence of outliers on the performance of methods for estimating accuracy in genomic prediction studies has barely been investigated to date. Heslot *et al.* (2013) examined the effect of entire outlying environments on genomic prediction, so their findings are difficult to compare with ours. As a result, there is a general dearth of comparable studies against which our findings can be reliably evaluated. Detection of outliers using standard diagnostic tools is well-known to be often far from trivial and less likely to be reliable because outliers can be present yet have inconspicuous residuals (Zewotir and Galpin 2005). Because the process of identifying and eliminating outliers prior to performing accuracy estimation in genomic prediction is only approximate, and because outliers can arise from multiple sources, including outlying individual observations, whole environments, or years, and therefore can be subtle and elusive to detect and eliminate, it is crucial to minimize potential adverse effects of any undetected outliers on the accuracy estimation in genomic prediction and on the reliability of selection decisions. To assess the impact of outliers on the performance of seven methods for estimating accuracy in genomic prediction based on RR-BLUP, we simulated 1000 replicate datasets according to an α-design for each of 10 scenarios distinguished by four configurations of genetic variances and sample sizes derived from two real maize datasets. The estimated predictive accuracy of each method on each dataset was evaluated against the simulated predictive accuracy and the accuracy estimated for the same dataset but contaminated, in turn, with one of three (scenarios 1 to 6) or two (scenarios 7 to 10) different outliers of increasing magnitudes.

### Heritability

Outliers strongly affected the accuracy with which heritability was estimated by all five methods used to estimate heritability regardless of scenario. The adverse influence of outliers on the estimated predictive accuracy of heritability increased with increasing magnitude of outliers regardless of sample size or genetic variance. However, the effect of outliers was not uniform and varied in both its character and severity across methods such that method 5, followed by methods 2 and 3, were the most robust to the influence of outliers in all scenarios. The adverse influence of outliers on the accuracy of heritability was also greater for the small than for the large datasets and became larger when the genetic variance was reduced irrespective of sample size. These results have three important practical implications for the design and choice of both the sample size and method to ensure reliable estimation of heritability in plant breeding studies. Specifically, studies designed to capture most of the phenotypic variance and that use datasets with large sample sizes and method 5 to estimate heritability are likely to yield the most accurate estimates of heritability.

### Predictive accuracy

Our comparative evaluation of the performance, stability, and robustness of the seven methods for estimating the accuracy of genomic prediction when phenotypic data are contaminated with outliers compared with when they are not (the benchmark) yielded many interesting and useful insights. Because the key assumptions, theoretical properties, computational efficiencies, and relative empirical performances of the seven methods under the same four configurations of genetic variances and sample sizes used to construct the 10 scenarios studied here were explored in depth by Estaghvirou *et al.* (2013), our prime focus here is specifically on how outliers modify the performance of these methods across the 10 simulated scenarios.

Estaghvirou *et al.* (2013) found estimates of predictive accuracy produced by method 5, which they proposed for estimating both heritability and predictive accuracy, and by method 7, which is widely used in animal breeding studies (Mrode and Thompson 2005; Henderson 1975; Van Raden 2008), to be the most accurate. In the present study, we found estimates of predictive accuracy for methods 5 and 7 to be the most stable and robust to the influence of outliers across all 10 scenarios we examined. Furthermore, methods 5 and 7 also gave broadly comparable estimates of predictive accuracy across all 10 scenarios. The other five methods were much more influenced by outliers across scenarios, portraying strong modification of their performances not only by the influences of outliers but also by genetic variance (and hence heritability) and sample size (number of genotypes).

Outliers had several evident detrimental effects on the performances of methods 1 to 4 and 6. These effects included a greater propensity to underestimate predictive accuracy and its variance when the sample size is small, overestimate predictive accuracy when the sample size is large, or for the mixed models to fail to converge to optimal parameter values, thus precluding computation of predictive accuracy. Increasing the size of outliers by a factor of 8/5 and 10/5 exacerbated these effects. The combination of large outliers and reduced genetic variance clearly aggravated the adverse influence of outliers on the estimated predictive accuracy and was associated with a far greater increase in the prevalence of undershooting, reduced incidence of overshooting, failure of the mixed models to iterate to convergence, and zero or extremely small estimated heritabilities. The composite effects on the estimated predictive accuracy of simultaneously increasing the magnitude of outliers and reducing the genetic variance were substantial regardless of sample size but were more debilitating when the sample size was small than when it was large.

For a given sample size, outliers substantially increased the time required by each of the seven methods to compute an estimate of predictive accuracy by prolonging the time needed to evaluate the log likelihood of the mixed models. This increase was especially marked for methods 1 to 4 and 6 that require cross-validation and therefore are more computationally expensive to implement than methods 5 and 7 that do not. Increasing the size of outliers (from 5 to 10) at a fixed sample size was associated with a further increase in computing time.

We have focused exclusively on genotypes for which phenotypic data are available. An extension to unphenotyped genotypes is thus highly desirable for applications in breeding programs. Such an extension is readily available for method 7 using standard procedures (Henderson 1984). A similar extension is currently lacking for method 5, but we are working on developing one to be presented in a future paper.

In conclusion, outliers had significant influences on the seven methods for estimating accuracy of genomic prediction, and these influences became more extreme when sample size and genetic variance were small. To ensure reliable estimation of accuracy in genomic prediction, it is therefore advisable to carefully check for and eliminate outliers whenever possible to design genomic selection studies to capture as much phenotypic variance as possible and yield large sample sizes. Because methods 5 and 7 are the simplest, computationally least expensive to implement, the most accurate and robust to outliers, small sample size, and genetic variance, using them to assess estimation accuracy in genomic prediction studies is decidedly preferable to using the five other competitor methods. Nevertheless, it is worth reiterating that we have considered only methods based on the RR-BLUP model, two sample sizes for genotypes, two sample sizes for markers, phenotypic data generated according to an α-design, and a single outlying observation in the phenotypic dataset. Further simulation studies that allow for wider variation in each of these five aspects would help establish the generality of our conclusions. We are currently exploring the consequences for accuracy estimation in genomic prediction of allowing for a greater range of variation in the number of genotypes and the number of markers and generating the phenotypic data according to different designs in follow-up studies.

## Supplementary Material

Supporting Information
