# Supplementary material for: Influence of Outliers on Accuracy Estimation in Genomic Prediction in Plant Breeding
Source: G3 (Bethesda). 2014 Oct 1;4(12):2317–28. doi: 10.1534/g3.114.011957 (PMC4267928; doi:10.1534/g3.114.011957)
Supplement: Supporting Information [file supp_4_12_2317__index.html]

Influence of Outliers on Accuracy Estimation in Genomic Prediction in Plant Breeding — Supporting Information 

# Influence of Outliers on Accuracy Estimation in Genomic Prediction in Plant Breeding

## Supporting Information for Ould Estaghvirou, Ogutu, and Piepho, 2014

**Files in this Data Supplement:**

- Supporting Information - Tables S1-S5 and Figures S1-S8 (PDF, 1 MB)
- Table S1 - The statistics, parameters and the symbols used to denote them in the text. (PDF, 112 KB)
- Table S2 - The variance components for the AgReliant real maize data set estimated by RR-BLUP models assuming genotypes are correlated according to the linear variance model. (PDF, 111 KB)
- Table S3 - The variance components for the KWS-Synbreed real maize data set estimated by RR-BLUP models assuming genotypes are correlated according to the linear variance model. (PDF, 111 KB)
- Table S4 - Descriptive statistics for the difference between the heritability estimated using datasets with (*r̂*2*g,ĝ,o*) and without (*r̂*2*g,ĝ*) outliers, taken as the benchmark, for the five methods (M1 to M5) in each of the 10 scenarios. (PDF, 114 KB)
- Table S5 - Descriptive statistics for the difference between the estimated predictive accuracy with (*r̂g,ĝ,o*) and without (*r̂g,ĝ*) outliers, taken as the benchmark, for the seven methods in each of the 10 scenarios. (PDF, 108 KB)
- Figure S1 - Studentized residuals for yield for the small data set (*n*=177 genotypes) contaminated with an outlier equal to five times the standard deviation of the residual error used to simulate the small datasets. (PDF, 162 KB)
- Figure S2 - Studentized residuals for yield for the large data set (*n*=698 genotypes) contaminated with an outlier equal to five times the standard deviation of the residual error used to simulate the large datasets. (PDF, 167 KB)
- Figure S3 - Studentized residuals for yield for the small data set (*n*=177 genotypes) contaminated with an outlier equal to 10 times the standard deviation of the residual error used to simulate the small datasets. (PDF, 154 KB)
- Figure S4 - Studentized residuals for yield for the large data set (*n*=698 genotypes) contaminated with an outlier equal to 10 times the standard deviation of the residual error used to simulate the large datasets. (PDF, 155 KB)
- Figure S5 - Studentized residuals for yield for the small data set (*n*=177 genotypes) contaminated with an outlier equal to eight times the standard deviation of the residual error used to simulate the small datasets. (PDF, 157 KB)
- Figure S6 - Frequency histograms of the deviations in the simulated true predictive accuracy *rg,ĝ* from the estimated predictive accuracy for the datasets with *r̂g,ĝ,o* (empty box and whiskers capped with brackets) and without *r̂g,ĝ* outliers, regarded as the benchmark (green boxes), for each of the seven methods in Scenarios 1 to 3. (PDF, 417 KB)
- Figure S7 - Frequency histograms of the deviations in the simulated true predictive accuracy *rg,ĝ* from the estimated predictive accuracy for the datasets with *r̂g,ĝ,o* (empty box and whiskers capped with brackets) and without *r̂g,ĝ* outliers, regarded as the benchmark (green boxes), for each of the seven methods in Scenarios 4 to 6. (PDF, 200 KB)
- Figure S8 - Frequency histograms of the deviations in the simulated true predictive accuracy *rg,ĝ* from the estimated predictive accuracy for the datasets with *r̂g,ĝ,o* (empty box and whiskers capped with brackets) and without *r̂g,ĝ* outliers regarded as the benchmark (green boxes) for each of the seven methods in Scenarios 7 to 10. (PDF, 209 KB)
