# Supplementary material for: Influence of Outliers on Accuracy Estimation in Genomic Prediction in Plant Breeding
Source: G3 (Bethesda). 2014 Oct 1;4(12):2317–28. doi: 10.1534/g3.114.011957 (PMC4267928; doi:10.1534/g3.114.011957)
Supplement: Supporting Information [file supp_g3.114.011957_011957SI.pdf]

G3· Genes | Genomes | Genetics

Influence of outliers on accuracy estimation in genomic prediction in plant breeding

Sidi Boubacar Ould Estaghvirou[†]; Joseph O. Ogutu[†*] and Hans-Peter Piepho[†]

[†]Bioinformatics Unit, Institute of Crop Science, University of Hohenheim

70599 Stuttgart, Germany.

[†]Bioinformatics Unit, Institute of Crop Science, University of Hohenheim,

Fruwirthstrasse 23, 70599 Stuttgart, Germany, +4971145923022, jogutu2007@gmail.com.

DOI: 10.1534/g3.114.011957

Table S1 The statistics, parameters and the symbols used to denote them in the text

**Statistic/parameter**	**Notation**
Sample standard deviation	s
Sample variance of the true genetic breeding values g	s_g^2
Sample correlation	r
Sample correlation between the BLUP of g and the observed "phenotypes" p	$r_{\hat{g},p}$
Sample true correlation between the true genetic breeding value g and the BLUP of g	$r_{g,\hat{g}}$
Sample covariance between the true and predicted breeding values	$s_{g,\hat{g}}$
Sample variance of predicted breeding value	$s_{\hat{g}}^2$
Phenotypic sample variance	s_p^2
Population standard deviation	σ
Population variance of the true genetic breeding values	σ_g^2
Population correlation	ρ
Population correlation between the true genetic breeding values g and observed "phenotypes" p	$\rho_{g,p}$
A sample assumed to have been selected from an infinite population of real or simulated genotypes	n

Table S2 The variance components for the AgReliant real maize data set estimated by RR-BLUP models assuming genotypes are correlated according to the linear variance model.

Variance components[†]	Estimate for Scenarios 1, 2 and 3	Estimate for Scenarios 4, 5 and 6
Genetic (σ_u^2)	0.2019	0.2019/10
Block (σ_b^2)	69.9089	69.9089
Residual (σ_e^2)	48.6728	48.6728

[†] Estimates for the other variance components are reported in Estaghvirou *et al*. (2013).

Table S3 The variance components for the KWS-Synbreed real maize data set estimated by RR-BLUP models assuming genotypes are correlated according to the linear variance model.

Variance components[‡]	Estimate for Scenarios 7 and 8	Estimate for Scenarios 9 and 10
Marker (σ_u^2)	0.005892	0.005892/10
Trial× Replicate × Block (σ_b^2)	6.3148	6.3148
Residual (σ_e^2)	53.8715	53.8715

[‡] Estimates for the other variance components are reported in Estaghvirou *et al*. (2013).

Table S4 Descriptive statistics for the difference between the heritability estimated using datasets with $\left(\hat{r}^2_{g,\hat{g},o}\right)$ and without $\left(\hat{r}^2_{g,\hat{g}}\right)$ outliers, taken as the benchmark, for the five methods (M1 to M5) in each of the 10 scenarios.

Scenario					#M1(10) $\hat{H}^2_{m_1}$	M2(11) $\hat{H}^2_{m_2}$	M3(12) $\hat{H}^2_{m_3}$	M4(13) $\hat{H}^2_{m_4}$	M5(15) $\hat{H}^2_{m_5}$
Nr	#Gen	Marker effect variance	Outliers	Statistics					
1	177	0.2019	5σ	Mean	-0.569^{c}	-0.463^{b}	-0.464^{b}	0.004^{a}	0.003^{a}
				Std	0.002	0.002	0.002	0.000	0.000
2	177	0.2019	8σ	Mean	-0.583^{d}	-0.443^{b}	-0.479^{c}	-0.002^{a}	-0.001^{a}
				Std	0.002	0.002	0.002	0.001	0.001
3	177	0.2019	10σ	Mean	-0.595^{d}	-0.439^{b}	-0.494^{c}	-0.008^{a}	-0.006^{a}
				Std	0.002	0.002	0.002	0.001	0.001
4	177	0.2019/10	5σ	Mean	-0.006^{a}	-0.009^{ab}	-0.011^{b}	-0.006^{a}	-0.016^{c}
				Std	0.001	0.001	0.001	0.000	0.001
5	177	0.2019/10	8σ	Mean	-0.012^{a}	-0.019^{b}	-0.022^{b}	-0.010^{a}	-0.029^{c}
				Std	0.001	0.002	0.002	0.001	0.002
6	177	0.2019/10	10σ	Mean	-0.016^{a}	-0.027^{b}	-0.031^{b}	-0.014^{a}	-0.038^{c}
				Std	0.001	0.002	0.002	0.001	0.002
7	698	0.005892	5σ	Mean	0.087^{c}	0.091^{b}	0.164^{a}	0.024^{e}	0.013^{e}
				Std	0.001	0.001	0.003	0.001	0.000
8	698	0.005892	10σ	Mean	0.075^{a}	0.076^{a}	0.074^{a}	0.011^{b}	0.006^{c}
				Std	0.001	0.001	0.001	0.001	0.000
9	698	0.005892/10	5σ	Mean	-0.004^{a}	-0.002^{a}	-0.004^{a}	-0.002^{a}	-0.004^{a}
				Std	0.000	0.000	0.000	0.000	0.000
10	698	0.005892/10	10σ	Mean	-0.011^{b}	-0.010^{b}	-0.011^{b}	-0.006^{a}	-0.014^{b}
				Std	0.001	0.001	0.001	0.000	0.000

For each of the 1000 datasets heritability was estimated using five methods for each scenario (1 to 10). Means for pairs of methods within each scenario with the same superscript letter are not significantly different at the 5% level of significance based on the *t*-test. #The number of the equation used in the text is in parenthesis. Methods 1 to 4 use cross-validation but Method 5 does not. σ is the standard deviation of the residual error. Note that positive differences denote overestimation whereas negative differences denote underestimation of the estimated heritability using datasets without outliers.

Table S5 Descriptive statistics for the difference between the estimated predictive accuracy with $\left(\hat{r}_{g,\hat{g},o}\right)$ and without $\left(\hat{r}_{g,\hat{g}}\right)$ outliers, taken as the benchmark, for the seven methods in each of the 10 scenarios.

Scenario	[†]Statistics	[§]M1(10)	M2(11)	M3(12)	M4(13)	M5(15)	M6(16)	M7(17)
1	Mean	0.002[a]	0.000[a]	0.000[a]	-0.009[a]	0.002[a]	-0.010[a]	0.002[a]
	Std	0.039	0.028	0.028	0.025	0.006	0.019	0.005
2	Mean	0.004[a]	-0.002[ab]	-0.002[ab]	-0.019[ab]	0.001[ab]	-0.025[b]	0.001[ab]
	Std	0.060	0.042	0.042	0.037	0.010	0.028	0.008
3	Mean	0.007[a]	-0.003[ab]	-0.003[ab]	-0.026[ab]	0.004[bc]	-0.037[c]	0.003[ab]
	Std	0.072	0.051	0.051	0.043	0.012	0.034	0.010
4	Mean	-0.127[c]	-0.095[b]	-0.095[b]	-0.184[d]	-0.017[a]	-0.176[d]	-0.015[a]
	Std	0.678	0.482	0.482	0.477	0.049	0.417	0.050
5	Mean	-0.177[c]	-0.133[b]	-0.133[b]	-0.246[e]	-0.028[a]	-0.212[d]	-0.026[a]
	Std	0.696	0.495	0.495	0.504	0.061	0.357	0.061
6	Mean	-0.179[c]	-0.135[b]	-0.135[b]	-0.272[e]	-0.038[a]	-0.225[d]	-0.036[a]
	Std	0.753	0.534	0.534	0.533	0.072	0.392	0.071
7	Mean	0.085[c]	0.081[c]	0.040[d]	0.143[b]	0.007[e]	0.196[a]	0.005[e]
	Std	0.030	0.030	0.061	0.038	0.009	0.037	0.006
8	Mean	0.084[c]	0.083[c]	0.084[c]	0.147[b]	0.004[d]	0.196[a]	0.003[d]
	Std	0.031	0.031	0.031	0.039	0.009	0.037	0.006
9	Mean	0.348[b]	0.354[b]	0.356[b]	0.460[a]	-0.003[c]	0.002[c]	-0.003[c]
	Std	0.591	0.598	0.602	0.087	0.004	0.016	0.003
10	Mean	0.347[b]	0.358[b]	0.355[b]	0.461[a]	-0.011[c]	0.002[c]	-0.009[c]
	Std	0.362	0.371	0.368	0.089	0.009	0.022	0.007

[†]Means for pairs of methods within each scenario with the same superscript letter are not significantly different at the 5% level of significance based on the *t*-test. [§]The number of the equation used in the text is in parenthesis. Scenarios are defined as in Table S4. Note that positive differences denote overestimation whereas negative differences denote underestimation of the predictive accuracy estimated using datasets without outliers.

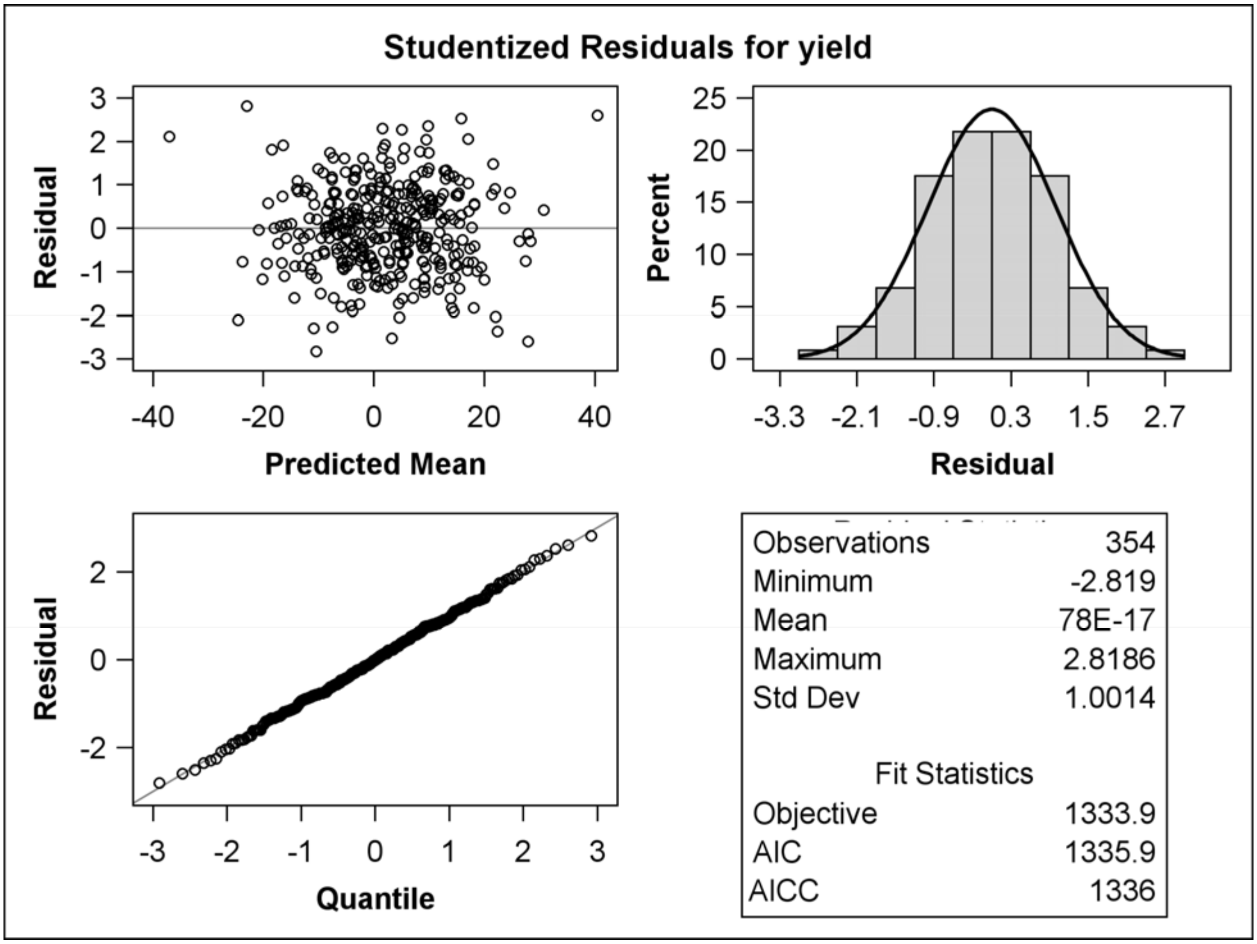

Figure S1 Studentized residuals for yield for the small data set (*n*=177 genotypes) contaminated with an outlier equal to five times the standard deviation of the residual error used to simulate the small datasets.

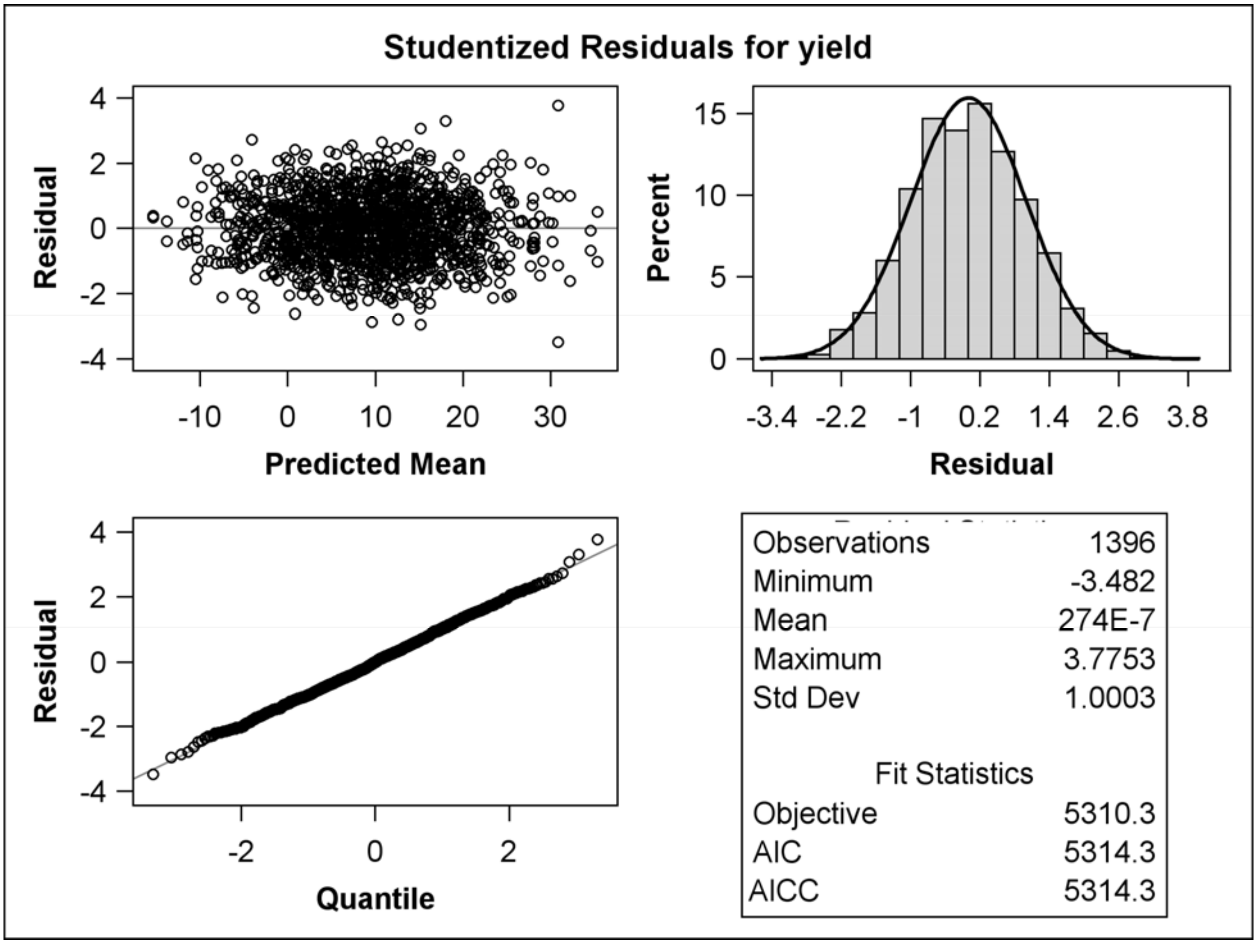

Figure S2 Studentized residuals for yield for the large data set (*n*=698 genotypes) contaminated with an outlier equal to five times the standard deviation of the residual error used to simulate the large datasets.

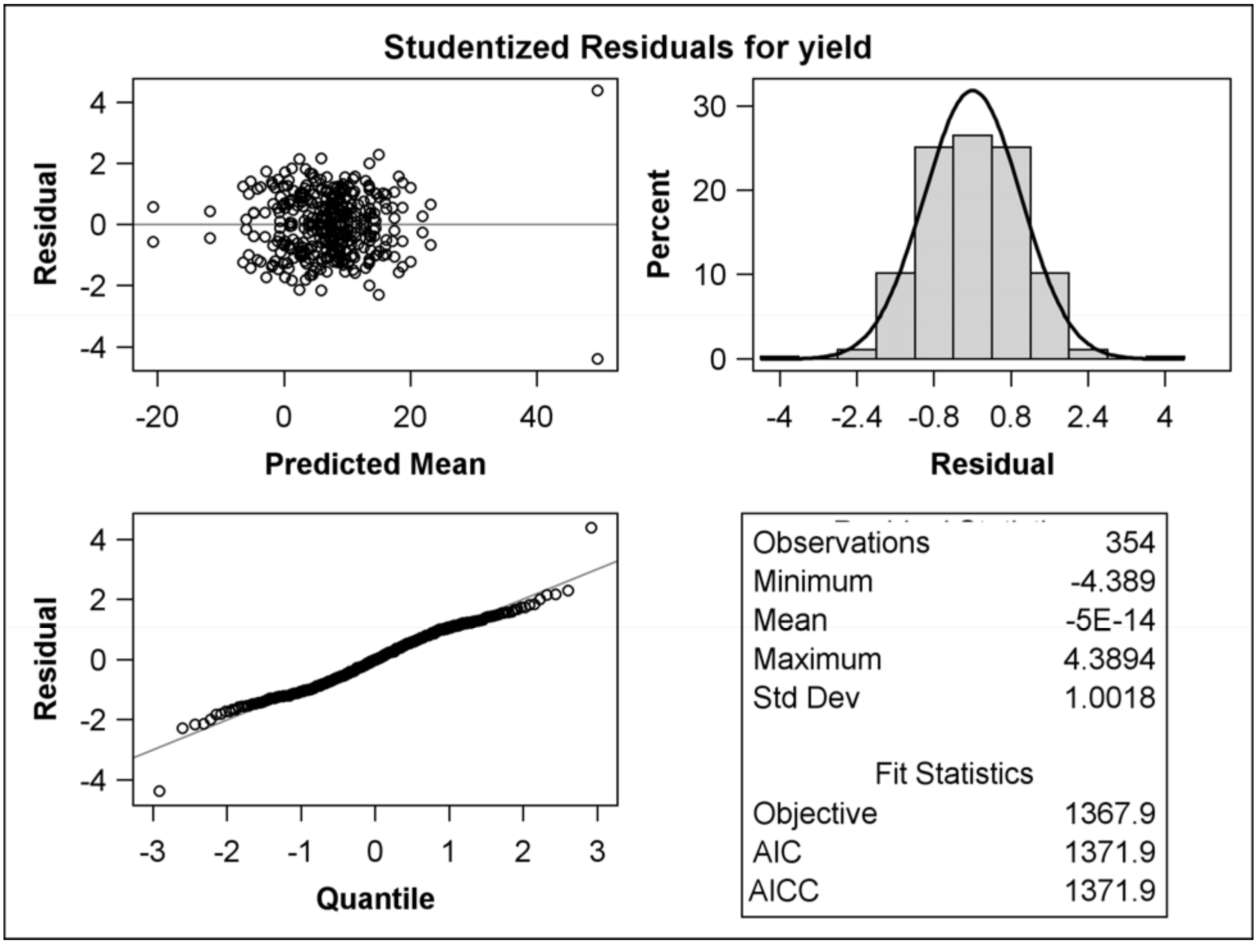

Figure S3 Studentized residuals for yield for the small data set (*n*=177 genotypes) contaminated with an outlier equal to 10 times the standard deviation of the residual error used to simulate the small datasets.

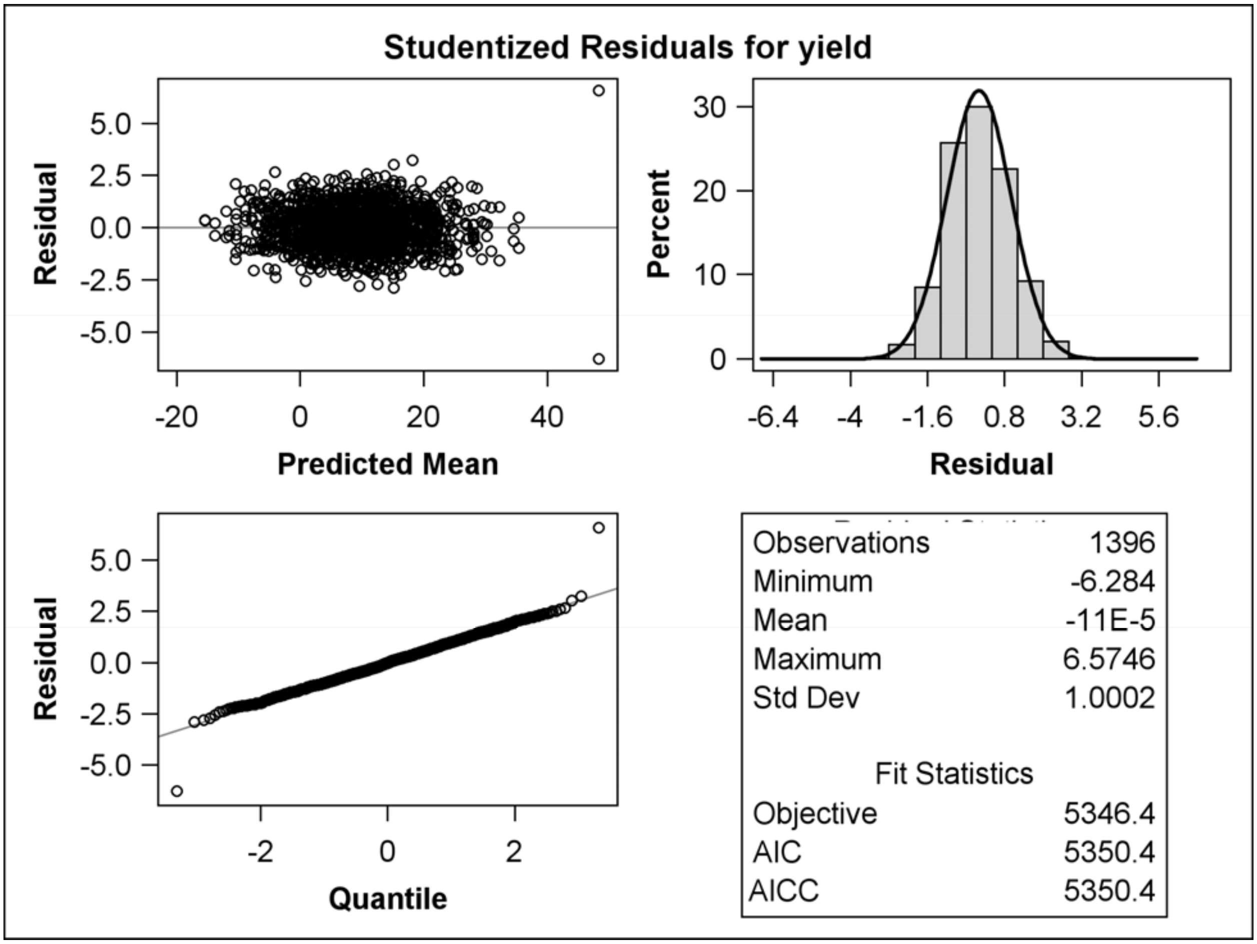

Figure S4 Studentized residuals for yield for the large data set (n=698 genotypes) contaminated with an outlier equal to 10 times the standard deviation of the residual error used to simulate the large datasets.

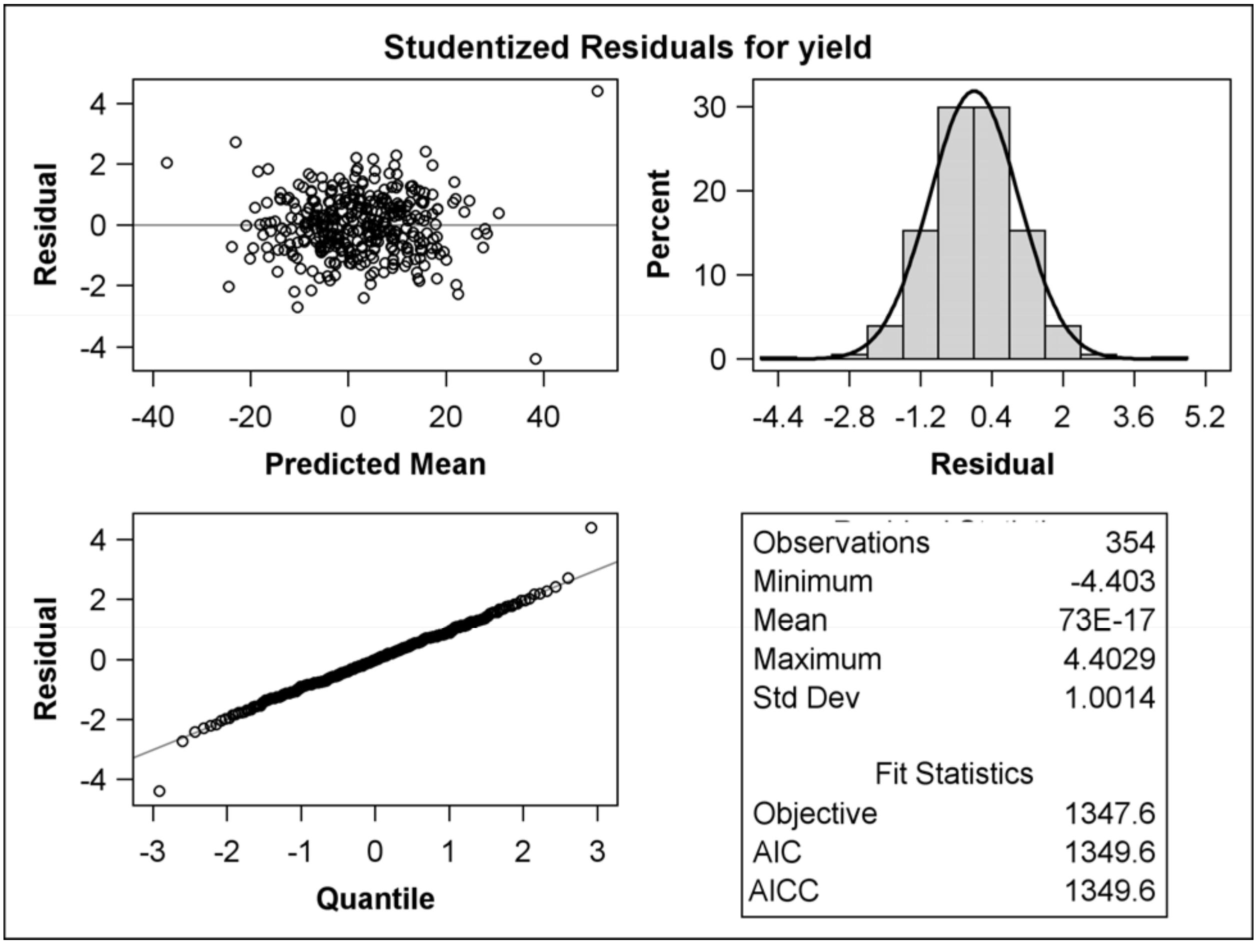

Figure S5 Studentized residuals for yield for the small data set (*n*=177 genotypes) contaminated with an outlier equal to eight times the standard deviation of the residual error used to simulate the small datasets.

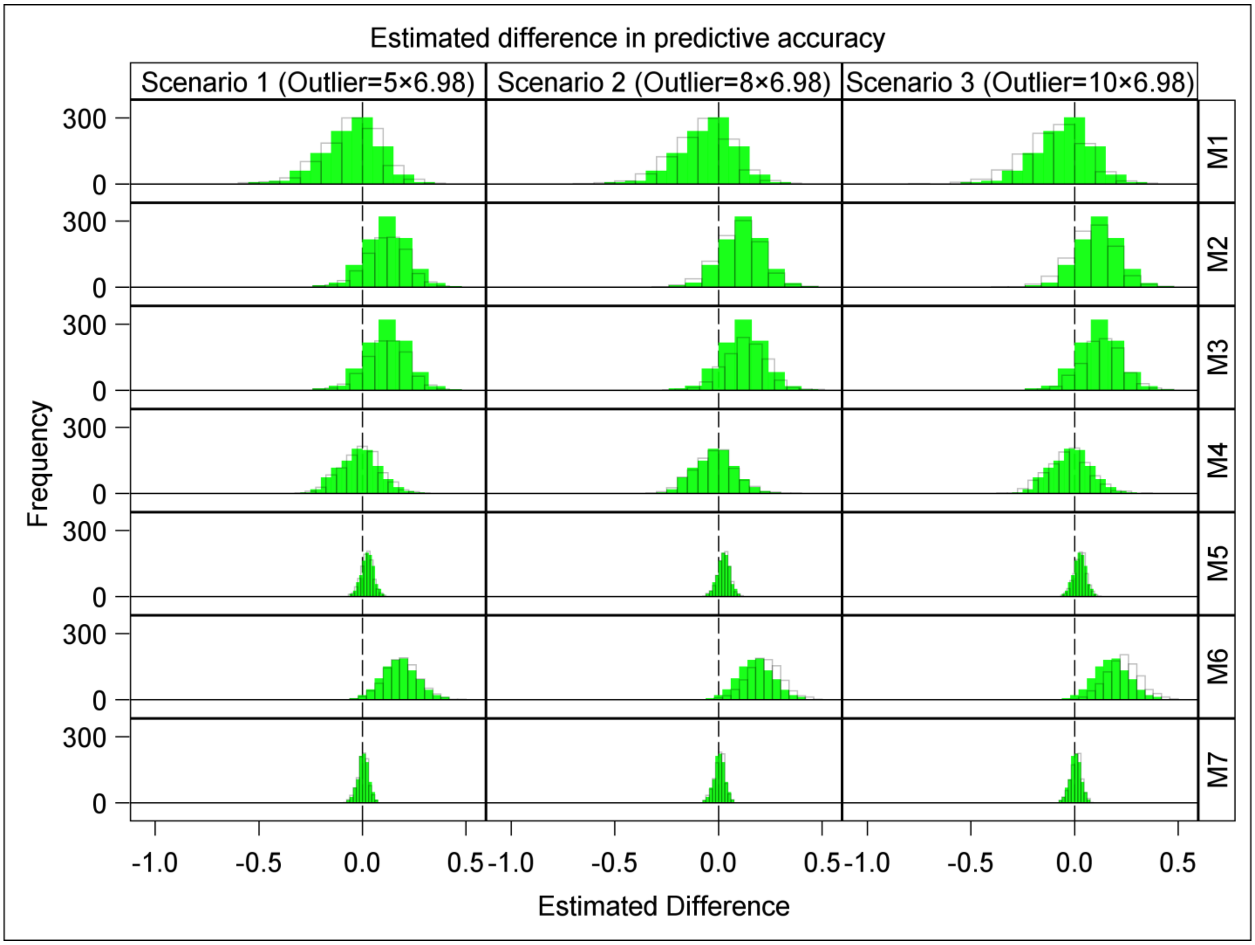

Figure S6 Frequency histograms of the deviations in the simulated true predictive accuracy $r_{g,\hat{g}}$ from the estimated predictive accuracy for the datasets with $\hat{r}_{g,\hat{g},o}$ (empty box and whiskers capped with brackets) and without $\hat{r}_{g,\hat{g}}$ (green boxes), outliers, regarded as the benchmark for each of the seven methods in Scenarios 1 to 3. All the scenarios are based on the same 1000 data sets simulated assuming 177 genotypes and a marker effect variance of 0.2019.

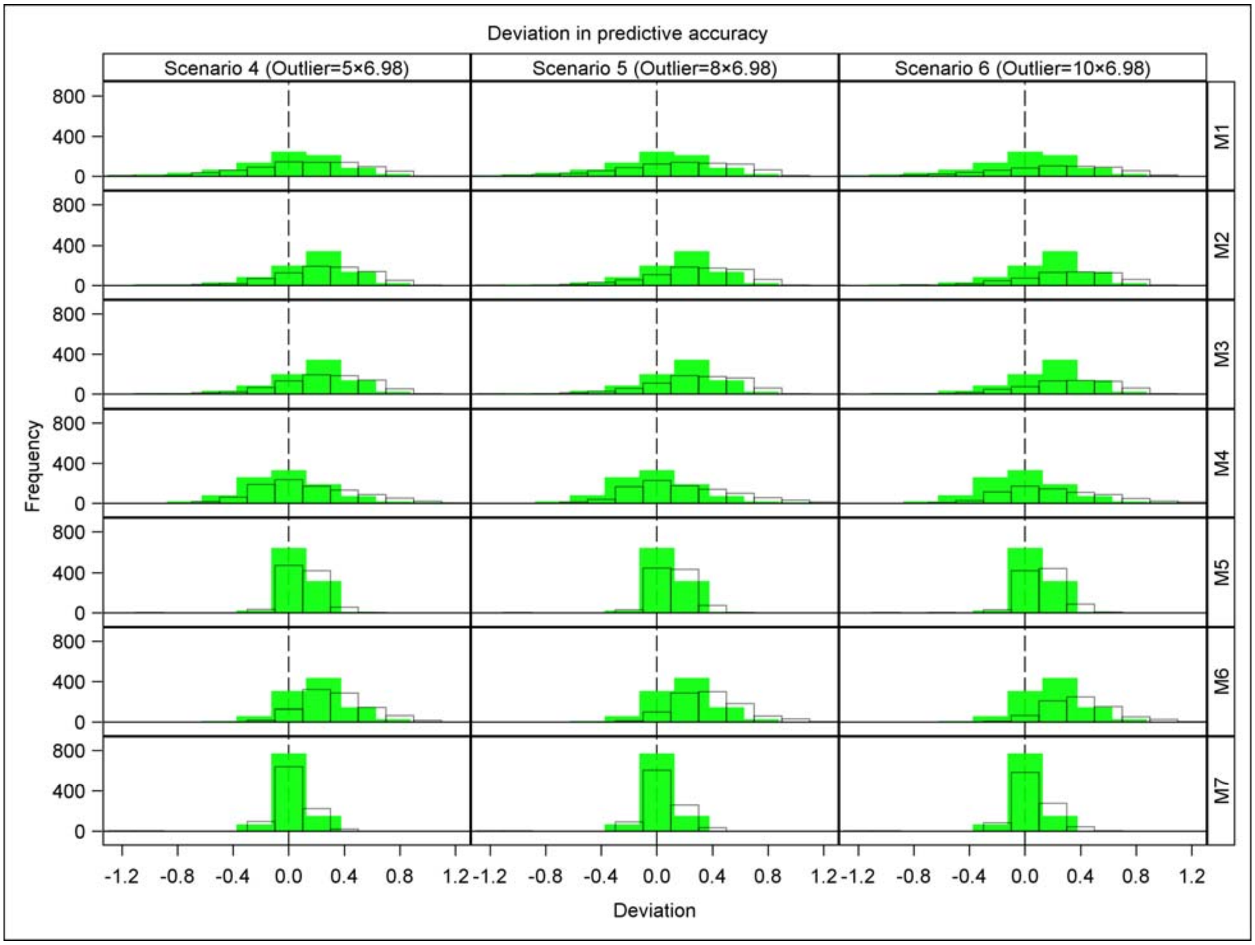

Figure S7 Frequency histograms of the deviations in the simulated true predictive accuracy $r_{g,\hat{g}}$ from the estimated predictive accuracy for the datasets with $\hat{r}_{g,\hat{g},o}$ (empty box and whiskers capped with brackets) and without $\hat{r}_{g,\hat{g}}$ outliers, regarded as the benchmark (green boxes), for each of the seven methods in Scenarios 4 to 6. All the scenarios are based on the same 1000 data sets simulated assuming 177 genotypes and a marker effect variance of 0.2019/10.

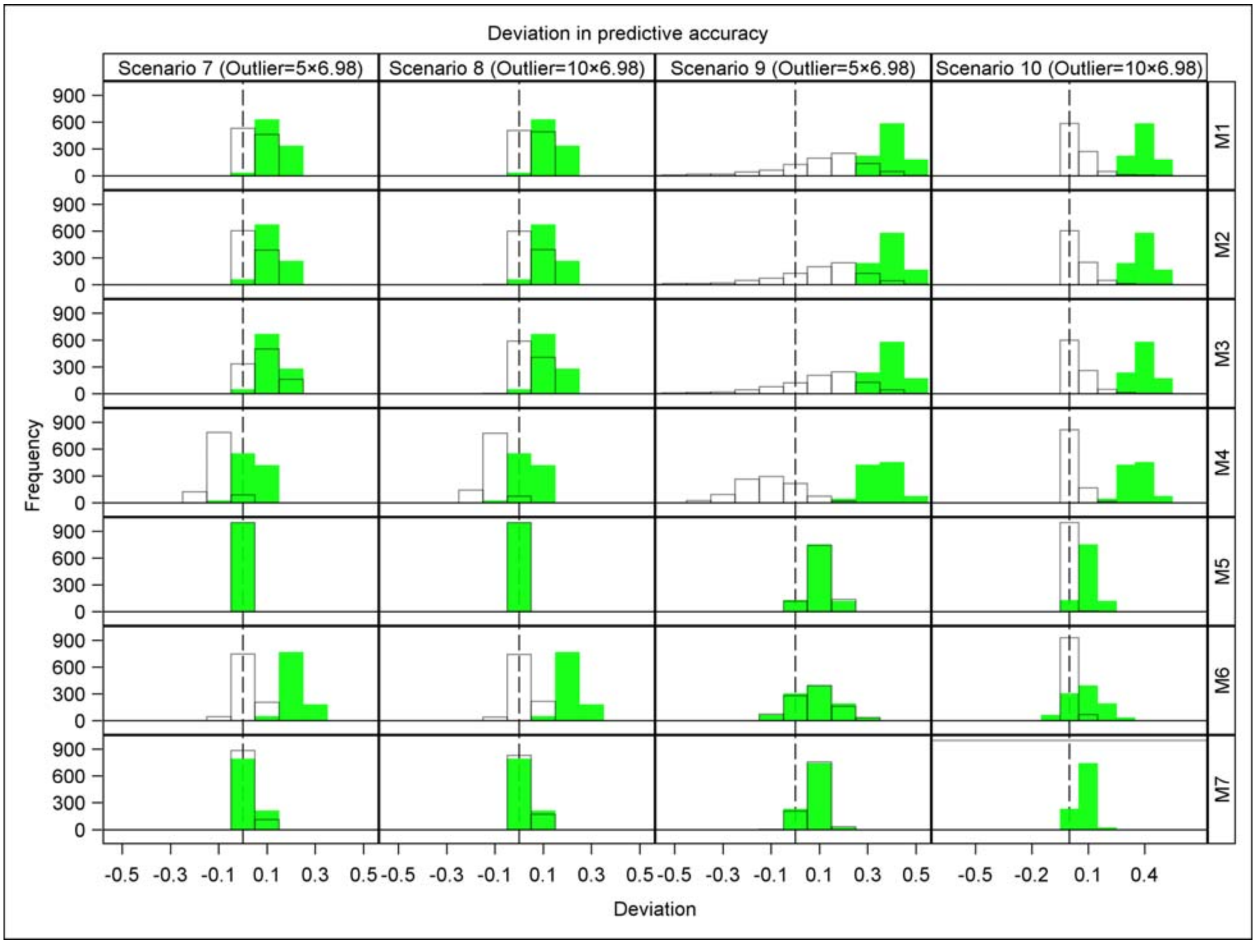

Figure S8 Frequency histograms of the deviations in the simulated true predictive accuracy $r_{g,\hat{g}}$ from the estimated predictive accuracy for the datasets with $\hat{r}_{g,\hat{g},o}$ (empty box and whiskers capped with brackets) and without $\hat{r}_{g,\hat{g}}$ outliers regarded as the benchmark (green boxes) for each of the seven methods in Scenarios 7 to 10. All the scenarios are based on the same 1000 data sets simulated assuming 698 genotypes and a marker effect variance of 0.005892 for Scenarios 7 and 8 and 0.005892/10 for Scenarios 9 and 10.
