# Supplementary material for: Influence of Outliers on Accuracy Estimation in Genomic Prediction in Plant Breeding
Source: G3 (Bethesda). 2014 Oct 1;4(12):2317–28. doi: 10.1534/g3.114.011957 (PMC4267928; doi:10.1534/g3.114.011957)
Supplement: Supporting Information [file supp_g3.114.011957_TableS1.pdf]

Table S1 The statistics, parameters and the symbols used to denote them in the text

**Statistic/parameter**	**Notation**
Sample standard deviation	s
Sample variance of the true genetic breeding values g	s_g^2
Sample correlation	r
Sample correlation between the BLUP of g and the observed "phenotypes" p	$r_{\hat{g},p}$
Sample true correlation between the true genetic breeding value g and the BLUP of g	$r_{g,\hat{g}}$
Sample covariance between the true and predicted breeding values	$s_{g,\hat{g}}$
Sample variance of predicted breeding value	$s_{\hat{g}}^2$
Phenotypic sample variance	s_p^2
Population standard deviation	σ
Population variance of the true genetic breeding values	σ_g^2
Population correlation	ρ
Population correlation between the true genetic breeding values g and observed "phenotypes" p	$\rho_{g,p}$
A sample assumed to have been selected from an infinite population of real or simulated genotypes	n
