# Supplementary material for: Influence of Outliers on Accuracy Estimation in Genomic Prediction in Plant Breeding
Source: G3 (Bethesda). 2014 Oct 1;4(12):2317–28. doi: 10.1534/g3.114.011957 (PMC4267928; doi:10.1534/g3.114.011957)
Supplement: Supporting Information [file supp_g3.114.011957_TableS2.pdf]

Table S2 The variance components for the AgReliant real maize data set estimated by RR-BLUP models assuming genotypes are correlated according to the linear variance model.

Variance components[†]	Estimate for Scenarios 1, 2 and 3	Estimate for Scenarios 4, 5 and 6
Genetic (σ_u^2)	0.2019	0.2019/10
Block (σ_b^2)	69.9089	69.9089
Residual (σ_e^2)	48.6728	48.6728

[†] Estimates for the other variance components are reported in Estaghvirou *et al*. (2013).
