# Supplementary material for: Influence of Outliers on Accuracy Estimation in Genomic Prediction in Plant Breeding
Source: G3 (Bethesda). 2014 Oct 1;4(12):2317–28. doi: 10.1534/g3.114.011957 (PMC4267928; doi:10.1534/g3.114.011957)
Supplement: Supporting Information [file supp_g3.114.011957_TableS3.pdf]

Table S3 The variance components for the KWS-Synbreed real maize data set estimated by RR-BLUP models assuming genotypes are correlated according to the linear variance model.

Variance components‡	Estimate for Scenarios 7 and 8	Estimate for Scenarios 9 and 10
Marker (σ_u^2)	0.005892	0.005892/10
Trial× Replicate × Block (σ_b^2)	6.3148	6.3148
Residual (σ_e^2)	53.8715	53.8715

‡ Estimates for the other variance components are reported in Estaghvirou *et al*. (2013).
