# Supplementary material for: Influence of Outliers on Accuracy Estimation in Genomic Prediction in Plant Breeding
Source: G3 (Bethesda). 2014 Oct 1;4(12):2317–28. doi: 10.1534/g3.114.011957 (PMC4267928; doi:10.1534/g3.114.011957)
Supplement: Supporting Information [file supp_g3.114.011957_TableS4.pdf]

Table S4 Descriptive statistics for the difference between the heritability estimated using datasets with $\left(\hat{r}^2_{g,\hat{g},o}\right)$ and without $\left(\hat{r}^2_{g,\hat{g}}\right)$ outliers, taken as the benchmark, for the five methods (M1 to M5) in each of the 10 scenarios.

Scenario					#M1(10) $\widehat{H}^2_{m_1}$	M2(11) $\widehat{H}^2_{m_2}$	M3(12) $\widehat{H}^2_{m_3}$	M4(13) $\widehat{H}^2_{m_4}$	M5(15) $\widehat{H}^2_{m_5}$
Nr	#Gen	Marker effect variance	Outliers	Statistics					
1	177	0.2019	5σ	Mean	-0.569[c]	-0.463[b]	-0.464[b]	0.004[a]	0.003[a]
				Std	0.002	0.002	0.002	0.000	0.000
2	177	0.2019	8σ	Mean	-0.583[d]	-0.443[b]	-0.479[c]	-0.002[a]	-0.001[a]
				Std	0.002	0.002	0.002	0.001	0.001
3	177	0.2019	10σ	Mean	-0.595[d]	-0.439[b]	-0.494[c]	-0.008[a]	-0.006[a]
				Std	0.002	0.002	0.002	0.001	0.001
4	177	0.2019/10	5σ	Mean	-0.006[a]	-0.009[ab]	-0.011[b]	-0.006[a]	-0.016[c]
				Std	0.001	0.001	0.001	0.000	0.001
5	177	0.2019/10	8σ	Mean	-0.012[a]	-0.019[b]	-0.022[b]	-0.010[a]	-0.029[c]
				Std	0.001	0.002	0.002	0.001	0.002
6	177	0.2019/10	10σ	Mean	-0.016[a]	-0.027[b]	-0.031[b]	-0.014[a]	-0.038[c]
				Std	0.001	0.002	0.002	0.001	0.002
7	698	0.005892	5σ	Mean	0.087[c]	0.091[b]	0.164[a]	0.024[e]	0.013[e]
				Std	0.001	0.001	0.003	0.001	0.000
8	698	0.005892	10σ	Mean	0.075[a]	0.076[a]	0.074[a]	0.011[b]	0.006[c]
				Std	0.001	0.001	0.001	0.001	0.000
9	698	0.005892/10	5σ	Mean	-0.004[a]	-0.002[a]	-0.004[a]	-0.002[a]	-0.004[a]
				Std	0.000	0.000	0.000	0.000	0.000
10	698	0.005892/10	10σ	Mean	-0.011[b]	-0.010 [b]	-0.011[b]	-0.006[a]	-0.014[b]
				Std	0.001	0.001	0.001	0.000	0.000

For each of the 1000 datasets heritability was estimated using five methods for each scenario (1 to 10). Means for pairs of methods within each scenario with the same superscript letter are not significantly different at the 5% level of significance based on the *t*-test. #The number of the equation used in the text is in parenthesis. Methods 1 to 4 use cross-validation but Method 5 does not. σ is the standard deviation of the residual error. Note that positive differences denote overestimation whereas negative differences denote underestimation of the estimated heritability using datasets without outliers.
