# Supplementary material for: Influence of Outliers on Accuracy Estimation in Genomic Prediction in Plant Breeding
Source: G3 (Bethesda). 2014 Oct 1;4(12):2317–28. doi: 10.1534/g3.114.011957 (PMC4267928; doi:10.1534/g3.114.011957)
Supplement: Supporting Information [file supp_g3.114.011957_TableS5.pdf]

Table S5 Descriptive statistics for the difference between the estimated predictive accuracy with $\left(\hat{r}_{g,\hat{g},o}\right)$ and without $\left(\hat{r}_{g,\hat{g}}\right)$ outliers, taken as the benchmark, for the seven methods in each of the 10 scenarios.

Scenario	†Statistics	§M1(10)	M2(11)	M3(12)	M4(13)	M5(15)	M6(16)	M7(17)
1	Mean	0.002[a]	0.000[a]	0.000[a]	-0.009[a]	0.002[a]	-0.010[a]	0.002[a]
	Std	0.039	0.028	0.028	0.025	0.006	0.019	0.005
2	Mean	0.004[a]	-0.002[ab]	-0.002[ab]	-0.019[ab]	0.001[ab]	-0.025[b]	0.001[ab]
	Std	0.060	0.042	0.042	0.037	0.010	0.028	0.008
3	Mean	0.007[a]	-0.003[ab]	-0.003[ab]	-0.026[ab]	0.004[bc]	-0.037[c]	0.003[ab]
	Std	0.072	0.051	0.051	0.043	0.012	0.034	0.010
4	Mean	-0.127[c]	-0.095 [b]	-0.095[b]	-0.184[d]	-0.017[a]	-0.176[d]	-0.015[a]
	Std	0.678	0.482	0.482	0.477	0.049	0.417	0.050
5	Mean	-0.177[c]	-0.133[b]	-0.133[b]	-0.246[e]	-0.028[a]	-0.212[d]	-0.026[a]
	Std	0.696	0.495	0.495	0.504	0.061	0.357	0.061
6	Mean	-0.179[c]	-0.135[b]	-0.135[b]	-0.272[e]	-0.038[a]	-0.225[d]	-0.036[a]
	Std	0.753	0.534	0.534	0.533	0.072	0.392	0.071
7	Mean	0.085[c]	0.081[c]	0.040[d]	0.143[b]	0.007[e]	0.196[a]	0.005[e]
	Std	0.030	0.030	0.061	0.038	0.009	0.037	0.006
8	Mean	0.084[c]	0.083[c]	0.084[c]	0.147[b]	0.004[d]	0.196[a]	0.003[d]
	Std	0.031	0.031	0.031	0.039	0.009	0.037	0.006
9	Mean	0.348[b]	0.354[b]	0.356[b]	0.460[a]	-0.003[c]	0.002[c]	-0.003[c]
	Std	0.591	0.598	0.602	0.087	0.004	0.016	0.003
10	Mean	0.347[b]	0.358[b]	0.355[b]	0.461[a]	-0.011[c]	0.002[c]	-0.009[c]
	Std	0.362	0.371	0.368	0.089	0.009	0.022	0.007

†Means for pairs of methods within each scenario with the same superscript letter are not significantly different at the 5% level of significance based on the *t*-test. §The number of the equation used in the text is in parenthesis. Scenarios are defined as in Table S4. Note that positive differences denote overestimation whereas negative differences denote underestimation of the predictive accuracy estimated using datasets without outliers.
