# Supplementary material for: Influence of Outliers on Accuracy Estimation in Genomic Prediction in Plant Breeding
Source: G3 (Bethesda). 2014 Oct 1;4(12):2317–28. doi: 10.1534/g3.114.011957 (PMC4267928; doi:10.1534/g3.114.011957)
Supplement: Supporting Information [file supp_g3.114.011957_FigureS5.pdf]

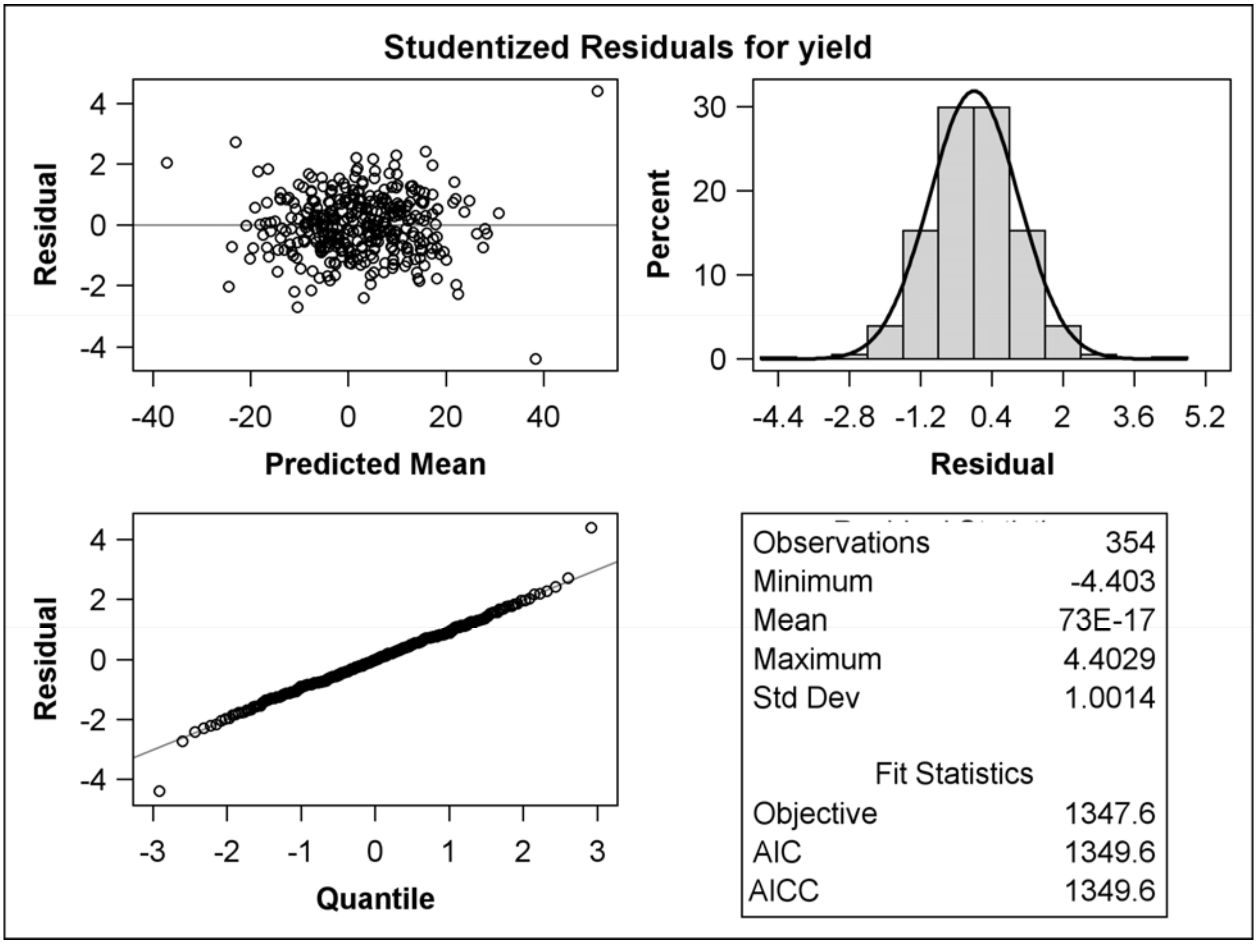

Figure S5 Studentized residuals for yield for the small data set (*n*=177 genotypes) contaminated with an outlier equal to eight times the standard deviation of the residual error used to simulate the small datasets.
