# Supplementary material for: Influence of Outliers on Accuracy Estimation in Genomic Prediction in Plant Breeding
Source: G3 (Bethesda). 2014 Oct 1;4(12):2317–28. doi: 10.1534/g3.114.011957 (PMC4267928; doi:10.1534/g3.114.011957)
Supplement: Supporting Information [file supp_g3.114.011957_FigureS7.pdf]

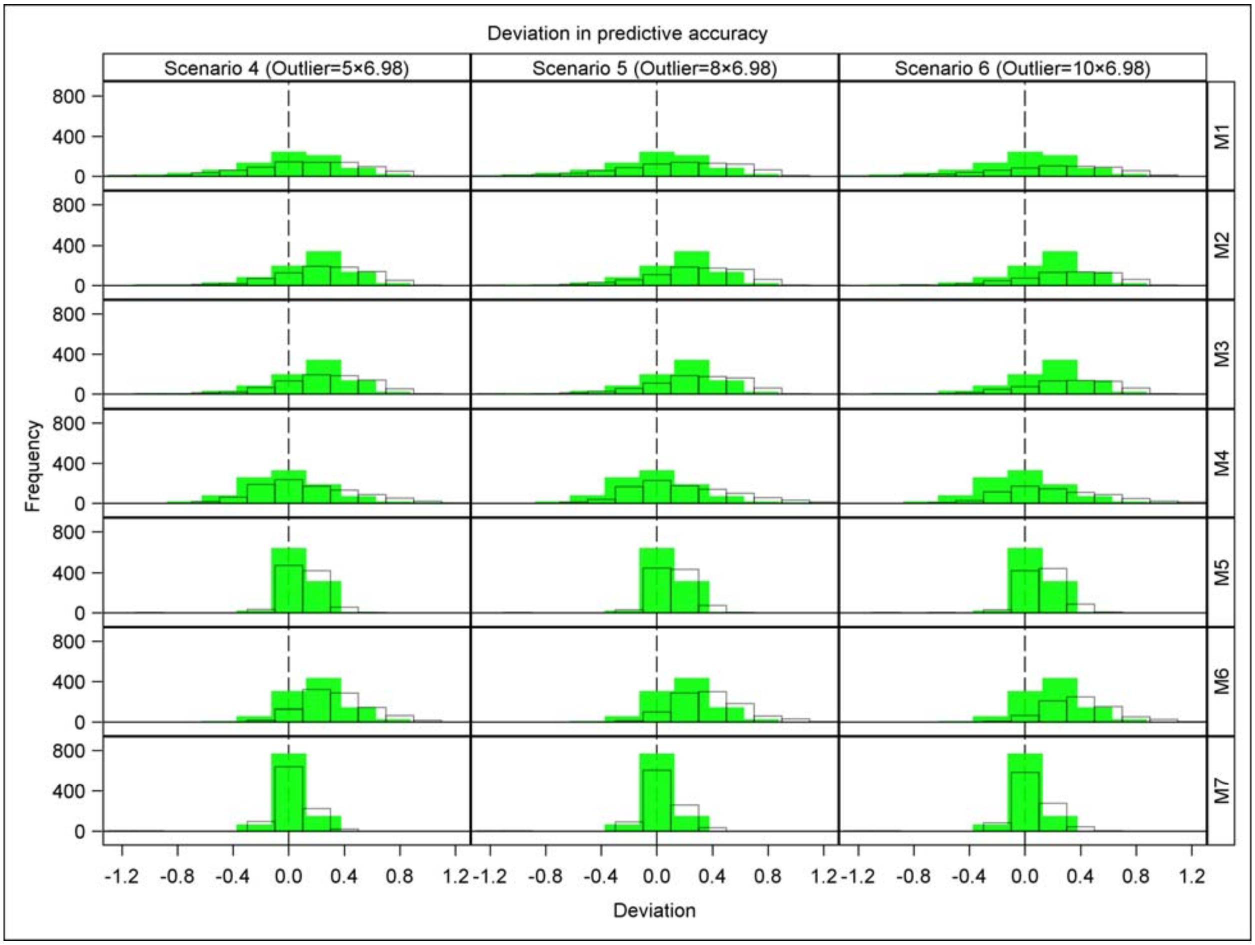

Figure S7 Frequency histograms of the deviations in the simulated true predictive accuracy $r_{g,\hat{g}}$ from the estimated predictive accuracy for the datasets with $\hat{r}_{g,\hat{g},o}$ (empty box and whiskers capped with brackets) and without $\hat{r}_{g,\hat{g}}$ outliers, regarded as the benchmark (green boxes), for each of the seven methods in Scenarios 4 to 6. All the scenarios are based on the same 1000 data sets simulated assuming 177 genotypes and a marker effect variance of 0.2019/10.
