# Supplementary material for: Influence of Outliers on Accuracy Estimation in Genomic Prediction in Plant Breeding
Source: G3 (Bethesda). 2014 Oct 1;4(12):2317–28. doi: 10.1534/g3.114.011957 (PMC4267928; doi:10.1534/g3.114.011957)
Supplement: Supporting Information [file supp_g3.114.011957_FigureS8.pdf]

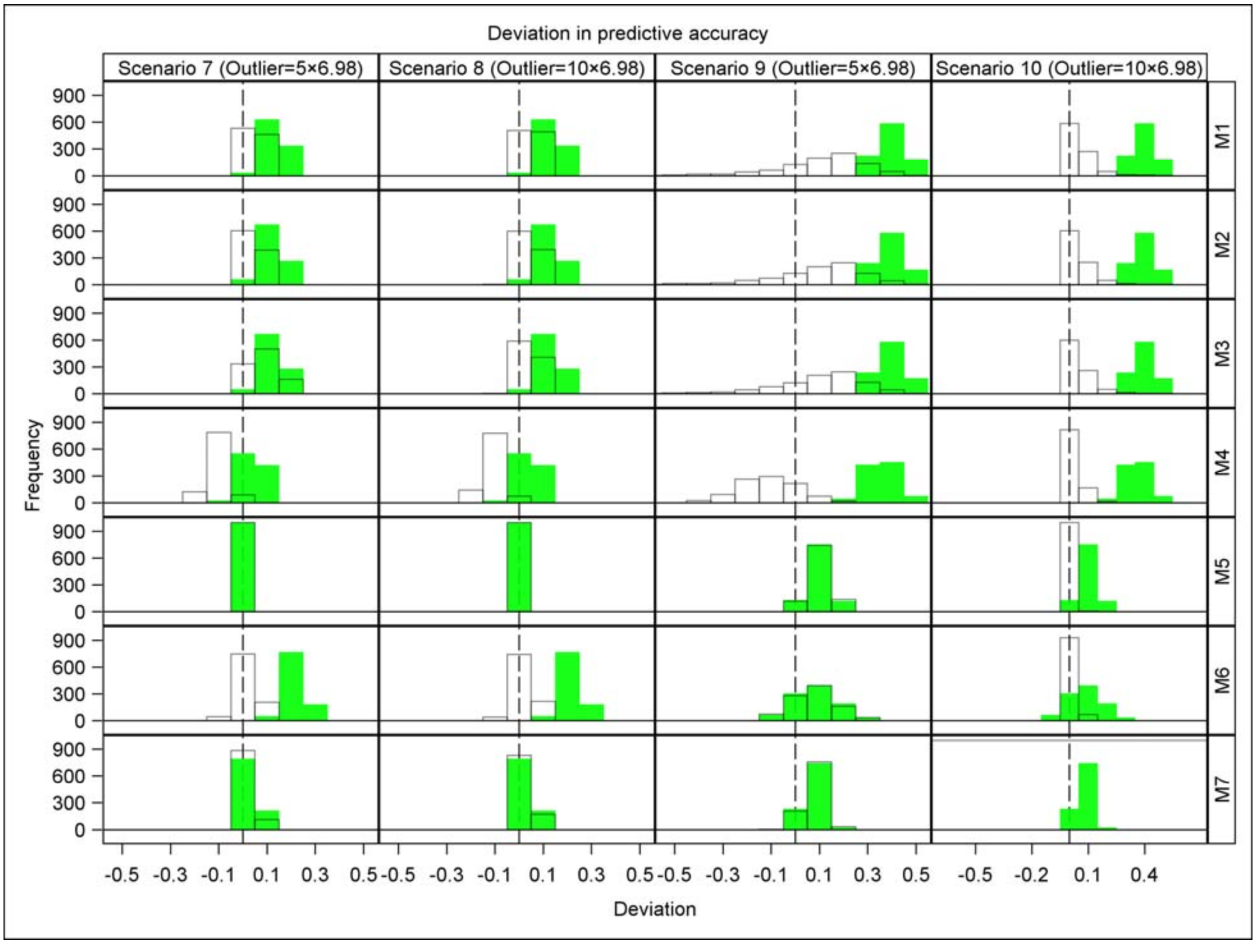

Figure S8 Frequency histograms of the deviations in the simulated true predictive accuracy $r_{g,\hat{g}}$ from the estimated predictive accuracy for the datasets with $\hat{r}_{g,\hat{g},o}$ (empty box and whiskers capped with brackets) and without $\hat{r}_{g,\hat{g}}$ outliers regarded as the benchmark (green boxes) for each of the seven methods in Scenarios 7 to 10. All the scenarios are based on the same 1000 data sets simulated assuming 698 genotypes and a marker effect variance of 0.005892 for Scenarios 7 and 8 and 0.005892/10 for Scenarios 9 and 10.
